# Comparative analysis of plastid genomes of non-photosynthetic Ericaceae and their photosynthetic relatives

**DOI:** 10.1038/srep30042

**Published:** 2016-07-25

**Authors:** Maria D. Logacheva, Mikhail I. Schelkunov, Victoria Y. Shtratnikova, Maria V. Matveeva, Aleksey A. Penin

**Affiliations:** 1 Lomonosov Moscow State University, A.N Belozersky Institute of Physico-Chemical Biology, Moscow, Russia; 2 Kazan Federal University, Institute of Fundamental Biology and Medicine, Kazan, Russia; 3 Institute for Information Transmission Problems, Russian Academy of Sciences, Moscow, Russia; 4 Lomonosov Moscow State University, Department of Bioengineering and Bioinformatics, Moscow, Russia; 5 Lomonosov Moscow State University, Department of Genetics, Moscow, Russia

## Abstract

Although plastid genomes of flowering plants are typically highly conserved regarding their size, gene content and order, there are some exceptions. Ericaceae, a large and diverse family of flowering plants, warrants special attention within the context of plastid genome evolution because it includes both non-photosynthetic and photosynthetic species with rearranged plastomes and putative losses of “essential” genes. We characterized plastid genomes of three species of Ericaceae, non-photosynthetic *Monotropa uniflora* and *Hypopitys monotropa* and photosynthetic *Pyrola rotundifolia*, using high-throughput sequencing. As expected for non-photosynthetic plants, *M. uniflora* and *H. monotropa* have small plastid genomes (46 kb and 35 kb, respectively) lacking genes related to photosynthesis, whereas *P. rotundifolia* has a larger genome (169 kb) with a gene set similar to other photosynthetic plants. The examined genomes contain an unusually high number of repeats and translocations. Comparative analysis of the expanded set of Ericaceae plastomes suggests that the genes *clpP* and *accD* that are present in the plastid genomes of almost all plants have not been lost in this family (as was previously thought) but rather persist in these genomes in unusual forms. Also we found a new gene in *P. rotundifolia* that emerged as a result of duplication of *rps4* gene.

Ericaceae is a large and diverse family of eudicots comprising approximately 120 genera and is divided into seven subfamilies^1^. Many species are economically important (crop and ornamental) plants. Most members of Ericaceae form associations with mycorrhizal fungi, on which they are dependent for such processes as nitrogen and phosphorus acquisition^2^. This dependence on fungi is most notable in species from the subfamily Monotropoidae, the members of which are partially or completely mycoheterotrophic, obtaining carbon through fungal interactions. Recent characterization of the plastid genomes of the photosynthetic Ericaceae *Vaccinium macrocarpon* and *Arbutus unedo*^3^,^4^ revealed many unique shared traits, such as a high repeat content, a drastic reduction in small single-copy regions, the putative pseudogenization of several essential genes (*accD*, *clpP*, *rps16*, *ycf1*, *ycf2*) and multiple rearrangements.

Here, we characterize the plastid genomes of two non-photosynthetic Ericaceae, *Monotropa uniflora* and *Hypopitys monotropa*, as well as that of their photosynthetic relative *Pyrola rotundifolia*. These are the first plastid genomes from mycoheterotrophic dicots reported to date, and the findings allow comparison with both parasitic dicots and mycoheterotrophic monocots. Among monocots, the most notable examples of mycoheterotrophy are orchids; indeed, virtually all members of this large and diverse family are dependent on fungi during germination, and some continue to gain carbon from fungi during their entire life cycle. It has been noted that mycoheterotrophic Ericaceae and Orchidaceae are very similar concerning their ecology^5^. The plastid genomes of a number of non-photosynthetic orchid species have been characterized, and the results have shown a wide spectrum of structures, from the early stages of degradation in *Corallorhiza*^6^ to the highly reduced genome in *Epipogium*^7^. Characterization of the *M. uniflora* and *H. monotropa* plastid genomes will reveal whether such parallelism exists at the molecular level with regard to the structure and mode of reduction of the plastome. Recently, DNA hybridization was applied to survey the gene content of plastid genomes in mycoheterotrophic Ericaceae (including *M. uniflora* and *H. monotropa*)^8^, with the results suggesting that photosynthesis-related genes have been lost from both species. However, DNA hybridization is not an exhaustive technique and can give both false positives (due to signals from nuclear or mitochondrial pseudogenes) and false negatives (due to the high divergence of plastid gene sequences).

A usually implicit assumption regarding the plastid genomes of non-photosynthetic plants is that their structure reflects the absence of selection acting on photosynthetic genes (thus leading to their loss or pseudogenization) and the retention of housekeeping genes. In this case, all non-photosynthetic plant plastomes should have a similar structure: highly reduced, with few genes responsible for housekeeping functions being retained. Indeed, a study of the highly reduced plastome of the orchid *Rhizanthella gardneri* revealed a striking similarity in gene content with the plastid genomes of unrelated parasitic plants^9^. However, with the increased availability of information on plastid genome sequences, it has become evident that the situation is more complex. Barrett and Davis^10^ proposed a model of gradual gene loss, in which photosynthesis-related genes are lost first and ribosomal RNA, ribosomal proteins, transfer RNAs and several housekeeping genes probably are among the last to be lost. This model is based primarily on orchid studies, and recent information on two highly reduced orchid genomes of *Epipogium* species^7^ appears to support it. Regardless, independent testing using data from unrelated taxa of mycoheterotrophic plants is required. The endpoint of plastid genome reduction in non-photosynthetic plants could be the complete loss of the plastome^11^. However, such cases seem to be extremely rare, only one is known in higher plants^12^. Retention of plastid genomes in photosynthetic plants is explained by a hypothesis which postulates that colocation of genes and their products in mitochondria and chloroplasts is beneficial for the redox regulation of gene expression (CoRR hypothesis, reviewed in ref. 13). However, this does not readily explain genome retention in non-photosynthetic plants; other hypotheses were put forward (reviewed in ref. 14). In order to assess their support by the data, more extensive sampling of non-photosynthetic plants is required.

To sum up, our study has two aims: first, to provide a more complete survey of gene content and plastome structure in Ericaceae, a group previously shown to have very unusual plastomes^3^,^4^, and second, focused on mycoheterotrophic Ericaceae, to complement the current knowledge on non-photosynthetic plant plastomes.

## Results and Discussion

### Structure and gene content of the *Pyrola rotundifolia* plastome

**Figure 1:**
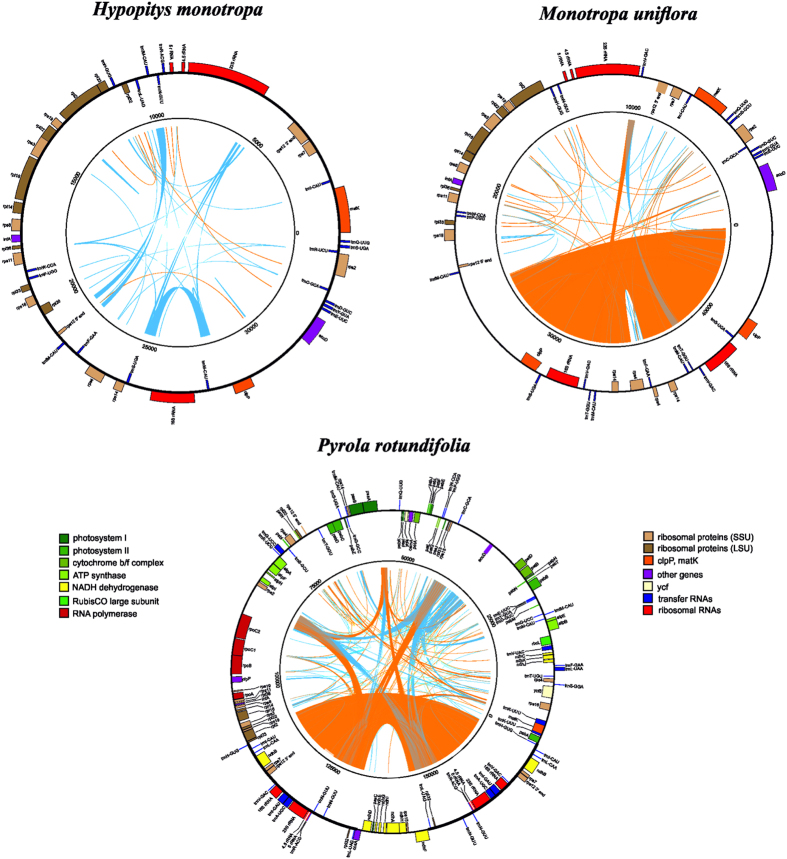
Plastid genome maps of *Pyrola rotundifolia*, *Monotropa uniflora* and *Hypopitys monotropa*. Genes shown inside the circle are transcribed clockwise; those outside the circle are transcribed counterclockwise. Blue and orange lines indicate direct and inverted repeats, respectively. The filled orange area indicates the inverted repeat region.

The plastid genome of *P. rotundifolia* is a circular molecule 168,995 bp in length. It exhibits a typical quadripartite structure: large and small single-copy (LSC and SSC) regions of 109,174 and 11,945 bp, respectively, and two parts of an inverted repeat (IR) of 23,937 bp each. The size of the small single-copy region is in contrast to what is observed in other Ericaceae, in which the small single-copy region is highly reduced, at ~3 kb (Fig. 1, Table 1). Additionally, the *P. rotundifolia* plastome is longer than that of most flowering plants due to an increased proportion of non-coding DNA represented by repeats and pseudogenes. The same is characteristic for *V. macrocarpon*^3^ but not for *A. unedo*, the plastome of which is ~150 kb^4^. The *P. rotundifolia* plastome contains 113 genes (considering duplicated genes as one gene), 20 of which are located in the IR. All of the photosynthesis-related genes usually found in the plastomes of photosynthetic angiosperms (encoding components of photosystems I and II, cytochrome b6/f complex, *rbcL*, ATP synthase complex) are present. There are, however, some deviations from a typical gene set: *ycf1* and *ycf2* are pseudogenes (both are present in the plastome as a small fragment, ~20% of intact gene length), and a novel gene combining the *rps4* N-terminal domain and transmembrane domains has evolved (see below). Three genes encoding components of the NADH dehydrogenase complex (*ndhD*, *ndhA*, *ndhF*) are represented by obvious pseudogenes (i.e., they are truncated and harbour frameshift mutations), and others (*ndhB*, *ndhC*, *ndhE*, *ndhH*, *ndhI*, *ndhJ*) contain an in-frame stop codon. This may indicate the early stages of pseudogenization or RNA editing; given that other genes of this group are undoubtedly pseudogenes, the second possibility is less probable. In other Ericaceae studied to date, these genes are either intact – as in *A. unedo*^4^, or only few (*ndhG*, *ndhI* and *ndhK*) are pseudogenes - as in *V. macrocarpon*^3^. The NDH complex is involved in chlororespiration and cyclic electron transport in photosystem I (reviewed in ref. 15) and is hypothesized to balance reactive oxygen species (ROS) levels to alleviate ROS-induced stress^16^. NDH genes are absent from non-streptophyte lineages of Archaeplastida, while present in most Streptophyta; together with their function this suggests that their retention in the plastome was the key adaptation to terrestrial way of life^17^. However, secondary loss of *ndh* genes from the plastome is known from several plant lineages (e. g., Geraniaceae, some gymnosperms, many orchids and some other monocot lineages). A phylogeny-wide transcriptome survey shows that this is a complete loss of the *ndh* gene complex, rather than the replacement of plastid genes by nuclear and/or mitochondrial genes^18^. Tobacco plants with knocked-out *ndh* genes are viable under optimal growth conditions but susceptible to water stress^19^,^20^, and mutations in *ndhF* were found to affect photosynthesis efficiency under changing light intensities^21^. These results suggest that the NDH complex plays a role in plant-environment interaction and that its dispensability depends on ecological factors (discussed, e.g. in ref. 22). Pseudogenization of *ycf2* and *ycf1*, mentioned above, is a very intriguing case. Both genes were found to be essential in tobacco^23^, but are absent in grasses (e.g. in ref. 24). Function of *ycf2* remains unknown; *ycf1* was recently identified as a component of the translocon on the inner envelope of chloroplasts^25^. This function was postulated based on experiments on *Arabidopsis thaliana*. The question of how universal the mechanism of protein translocation involving *ycf1* is disputable^26^,^27^. de Vries *et al.*^26^ note that the loss of *ycf1* usually co-occurs with the loss of *accD*. Ericaceae seemed to support this observation. However, we argue that *accD* is intact at least in several Ericaceae (see below). As for *ycf1*, *ycf1*-like sequence in the *P. rotundifolia* plastome contains a truncated ORF (~200 amino acids) and is thus unlikely to be functional. The same is true for two other photosynthetic Ericaceae – *V. macrocarpon* and *A. unedo*.

### Structure and gene content of the *Monotropa uniflora* and *Hypopitys monotropa* plastomes

In both species, the plastid genome is highly reduced, at ~46 kb in *M. uniflora* and 35 kb in *H. monotropa*; the difference in length is because the latter lacks the IR, whereas the former retains an IR of ~9.5 kb (Fig. 1, Table 1). As in *A. unedo* and *V. macrocarpon*, *M. uniflora* has a very short small single-copy region of ~800 bp which encodes a single gene, *trnF-GAA*. The GC-content is 34.4% in *H. monotropa* and 29.4% in *M. uniflora*. The latter is much less than observed in other plants and is due to the presence of numerous AT-rich repeats (see below) and to an AT-bias in protein-coding sequences. As expected, the gene set is highly reduced in both species, with all genes encoding components of the photosynthetic apparatus having been lost or pseudogenized (Table 2). Genes encoding a bacterial type RNA polymerase (PEP – plastid encoded polymerase) subunits were also lost; the same is observed in most non-photosynthetic plants except for the liverwort *Aneura mirabilis*^28^. Although in model plants PEP is active across the whole organism, including non-green parts, its main function is the transcription of photosynthesis-related genes, while housekeeping genes are transcribed by another, nuclear encoded polymerase (NEP)^29^. Thus PEP function is dispensable in non-photosynthetic plants (see, e.g. ref. 30). Notably, ATP synthase genes are also lost in both species (in *H. monotropa*, *atpB* is present as pseudogene) while in many other non-photosynthetic plants they are retained^6^,^31^,^32^,^33^. Recently Kamikawa *et al.*^34^ proposed that the ATP synthase complex is necessary for the function of TAT (twin-arginine translocator) system which translocates proteins into thylakoids. The characterization of *H. monotropa* and *M. uniflora* transcriptome will show whether this system is active in these plants. In view of Kamikawa *et al.*^34^ hypothesis, we expect that it is not. The only class of genes not influenced by the reduction is ribosomal RNA genes; most ribosomal protein genes are also intact. Despite the longer length of the *M. uniflora* plastome, it contains fewer genes than *H. monotropa*. Of nine genes encoding ribosomal proteins for the large subunit, which are generally present in plastid genomes (reviewed in ref. 35), two (*rpl32* and *rpl23*) have been lost from *M. uniflora*; in contrast, *H. monotropa* encodes a complete set. In both species, the status of *rpl20* is unclear; compared to *A. unedo* it has a premature stop codon which results in ~70 bp shorter ORF. In *V. macrocarpon*, *rpl20* ORF is present (though not annotated) and is 42 nt shorter than in *A. unedo*. Regarding small subunit ribosomal RNA proteins, two (*rps16* and *rps15*) have been lost from both species. *rps16* is known to be lost from the plastome and functionally replaced by nucleus-encoded copy in several photosynthetic plants^36^, and *rps15* is non-essential, according to knock-out studies^35^. In addition, the number of transfer RNA genes is reduced compared to photosynthetic plants. Notably, all intron-containing tRNA genes are absent in both species, though intron-containing protein-coding genes (*rpl2*, *rpl16*) are intact. Despite a larger size, *M. uniflora* plastome has a more reduced set of tRNA genes: in addition to the above-mentioned genes *trnG-GCC*, *trnL-CAA*, *trnL-UAG*, *trnR-ACG*, *trnR-UCU*, *trnS-GGA* and *trnT-UGU* are also absent and *trnV-GAC* and *trnM-CAU* are pseudogenes (they maintain conserved tRNA secondary structure but exhibit several nucleotide substitutions, including in the anticodon). *H. monotropa* possesses intact *trnL-UAG*, *trnM-CAU*, *trnR-ACG* and *trnR-UCU* genes. Remarkably, in both species *trnE-UUC* – the gene that is hypothesized to be the main reason for plastome conservation because of its necessity for heme biosynthesis^14^ - is intact. *matK* is present but *trnK* intron and exons are lost; *infA* is intact. *ycf1* and *ycf2* are absent in both species. *accD* and *clpP* genes persist but are highly diverged (see details below).

Both species display a gene order that deviates from the typical and from those of *A. unedo* and *V. macrocarpon*. The most surprising finding is that the ribosomal RNA operon, which is highly conserved in its structure and position and is located in the inverted repeat region in most plants^37^, is divided into two parts. The organization of rRNA genes into a single operon facilitates their coordinated expression, which is important for plastid ribosome biogenesis. However, in *M. uniflora*, *rrn16* is located in the IR, together with a few other genes (*rps4*, *rps14*, *trnS-UGA,* and *accD*), and other rRNA genes are located in the LSC region. *rrn16* is also located distantly from other rRNA genes in *H. monotropa*. Experimental data show that the rRNA operon can be transcribed by both nucleus- and plastid-encoded RNA polymerases (reviewed in ref. 37), which are presumably differentially utilized during plant development (e.g. in ref. 38). Although the operon is structurally stable, its promoter region is labile; for example, parasitic plants from the genus *Cuscuta* lacking the PEP enzyme also have lost PEP promoter motifs^30^. As both *rrn16* and *rrn23* are expressed in *H. monotropa* (unpublished data), we suggest that the *rrn23*-*rrn4.5*-*rrn5* gene cluster has acquired a new NEP promoter. The largest block that is colinear with the plastomes of other plants is that containing most ribosomal protein genes: the S10 operon.

As mentioned above, the gene contents of *M. uniflora* and *H. monotropa* were previously assessed using DNA hybridization^8^. Although the results are largely consistent, there are a few exceptions, e.g., hybridization indicated the presence of several *ndh* and RNA polymerase genes, which we demonstrated to be absent from the plastome. Presumably, this discrepancy can be explained by the presence of plastid-derived sequences in the mitochondrial or nuclear genome, which may generate positive signals in hybridization experiments.

When this article was in review, a note describing the plastid genome of *Hypopitys monotropa* was published^39^. It is mostly congruent with our findings on *H. monotropa* plastome in terms of length and gene content (with the exception of two genes – *accD* and *clpP* - that will be discussed in detail below) but the gene order is different. Also, the sequences are highly divergent (10% for protein coding genes at amino acid level) indicating intraspecific polymorphisms or the existence of cryptic species.

### Repetitive sequences in Ericaceae plastomes

We explored the repeat contents of five Ericaceae plastomes and found a large increase of repetitive DNA fraction in all species, regardless of their photosynthetic capacity. The results for *V. macrocarpon* and *A. unedo* are consistent with previous reports^3^,^4^. The highest fraction of repeats was found in *P. rotundifolia* (Supplementary Table 1) and *V. macrocarpon*. The *V. macrocarpon* and *P. rotundifolia* repeats differ in sequence, are located at different sites and are not homologous, emphasizing the tendency of Ericaceae to accumulate repeats. In *P. rotundifolia*, the longest repeats originate from a series of tandem duplications in the *accD* gene (see discussion below), and the plastome region containing the *accD* gene in *M. uniflora* also harbours multiple repeats (Supplementary Fig. 1). The entire region is extremely AT-rich and of low DNA complexity. Similarly, abundant AT-rich repeats were recently reported in a phylogenetically unrelated plant, the orchid *Cypripedium japonicum*^40^. These sites have proven to be informative markers for studies of population genetics in this species, and *M. uniflora* repeats are potentially useful for this purpose too.

### GC content and codon usage in Ericaceae plastomes: contrasts between two non-photosynthetic species

**Figure 2:**
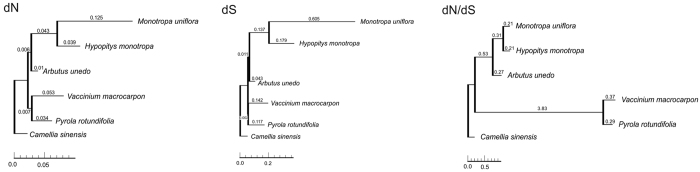
Phylogenetic tree of Ericaceae reconstructed based on plastid rDNA, plastid protein-coding and nuclear rDNA sequences. Branch lengths correspond to dN, dS and dN/dS values. The high dN/dS (3.83) value for *Pyrola*/*Vaccinium* branch is presumably an artefact caused by an insufficient number of synonymous substitutions.

Previous studies of the plastomes of non-photosynthetic plants suggest that they exhibit the following tendencies with regard to the molecular evolution of their protein-coding genes: high AT-content, exhibiting in the most extreme cases codon and amino acid usage bias^7^,^9^,^20^; increased rates of nucleotide substitutions without relaxation of selection in housekeeping genes^7^,^21^; and neutral evolution of photosynthesis-related genes.^6^,^7^,^9^ Furthermore, recent studies of highly reduced plastomes^7^,^41^,^42^,^43^ suggest that the degree of manifestation of these tendencies correlates with the degree of plastome reduction. Thus, we expected to observe these features in *H. monotropa* and *M. uniflora*, which have highly reduced plastomes. However, our comparative analysis revealed strong evidence for such patterns only in *M. uniflora*; in *H. monotropa*, the patterns are more similar to those observed in photosynthetic Ericaceae. The total GC content of photosynthetic Ericaceae members ranges between 36 and 38%, which is typical for most plants. In *H. monotropa* it is only slightly lower, and notably lower in *M. uniflora* (Table 3). In general, the most GC-rich region of the plastome is the IR. The *M. uniflora* IR shows the lowest GC content due to the presence of a highly AT-rich repetitive region in *accD* and the unusual localization of *rrn23* outside the IR. With regard to codon usage, we found no apparent alterations in *H. monotropa* compared to photosynthetic Ericaceae, though *M. uniflora* seems to be biased toward AT-rich codons (Supplementary Table 2). Rates of substitution accumulation in shared protein-coding genes differ significantly between all pairs of the studied Ericaceae (p-value < 10^−3^ by Tajima’s relative rate test with Bonferroni correction). The rates of substitutions in non-synonymous and synonymous positions change proportionally, so the dN/dS ratio is similar in all branches, except for two very short branches, where it is hard to estimate (Fig. 2). The rate of substitution accumulation is highest in a lineage of *M. uniflora*. In a lineage of *H. monotropa* it is closer to the values in photosynthetic lineages (Fig. 2, Supplementary Table 3). As mentioned above, the *M. uniflora* plastome exhibits an unusually high number of low-complexity AT-rich repeats, and as in *C. japonicum*^40^, these abundant AT-rich repeats might serve as markers for population genetic analyses in *Monotropa*.

### Pseudogenes in Ericaceae plastomes

Pseudogenes are quite rare in plant plastomes; a few notable exceptions are most non-photosynthetic plants and several green plants (for review see ref. 44). Among the latter, the most prominent and well-studied are Geraniaceae; these plants contain highly rearranged plastomes, in which many regions are duplicated and additional copies of genes arise from these duplications^45^,^46^. Similar events have occurred in *Trachelium* (Campanulaceae)^47^. In both cases, the genome duplications are correlated with a high repeat content and an increased substitution rate.

Similar to the abovementioned plants, species of Ericaceae, photosynthetic or not, possess an unusually large number of pseudogenes (Table 2). These pseudogenes can be divided into three categories: (1) pseudogenes of photosynthesis-related genes in non-photosynthetic Ericaceae; (2) presumably non-functional copies of intact genes; and (3) pseudogenes of genes that are dispensable or became dispensable due to the presence of a functional copy in the nuclear genome. In contrast to non-photosynthetic plants in the early stages of plastome reduction, which retain pseudogenes for most of photosynthesis-related genes (e.g. refs 28,32) pseudogenes of the first category are present only in *H. monotropa* (*atpB*, *psaB*, *ccsA*) but have been completely lost from *M. uniflora*. Pseudogenes resulting from the degradation of duplicated genes are numerous in *P. rotundifolia* and *V. macrocarpon* (*psaA*, *rps18*, *rps3* in *V. macrocarpon*^3^ and *rrn16*, *rpl2*, *petD* and *accD* in *P. rotundifolia*) while in *A. unedo* the only example is *ndhA*^4^. Notably, *P. rotundifolia* and *V. macrocarpon* have the highest repeat content among Ericaceae (Supplementary Table 1). Repeats are known to favour structural changes, including duplications, by the mechanism of illegitimate recombination^48^. We hypothesize that this mechanism gave rise to the above-mentioned pseudogenes. The third category includes *ycf1*, *ycf2* and several *ndh* genes. However, the reason for the pseudogenization of genes in this category (either dispensability or functional replacement) is less clear because it should ideally be inferred from genome-wide data on the presence of genes in the nuclear genome and on functional genetic studies. One of the few examples of such a study thus far is that of Rousseau-Gueutin and coworkers^49^, who explored the replacement of plastid *accD* in Campanulaceae by a nuclear gene arising via a transfer from the plastome.

### Unusual genes in Ericaceae plastid genomes

**Figure 3:**
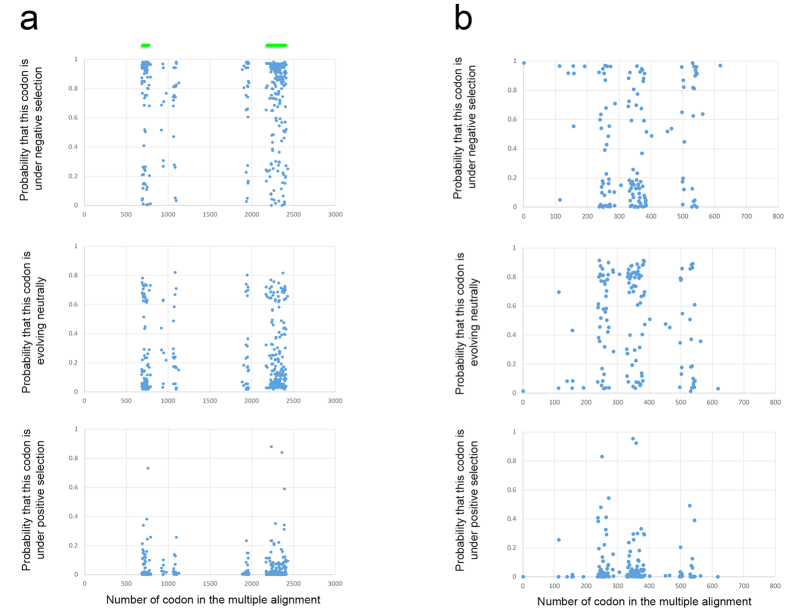
Mode of selection for *accD* (**a**) and *clpP* (**b**) in Ericaceae. The dots indicate the probability of negative selection, neutral evolution and positive selection for codons in genes *accD* (**a**) and *clpP* (**b**). The probabilities are estimated by the Empirical Bayes method in PAML. Green lines above the *accD* diagram designate regions homologous to parts of *accD* from plants with typical *accD* structure.

Although many genes in Ericaceae have been pseudogenized, a close examination of two (*accD* and *clpP*) that were previously reported to be pseudogenes shows that they may indeed represent intact genes. *clpP* encodes a subunit of the clp-protease complex, which is necessary for removing undesirable proteins within plastids (reviewed in ref. 50). *accD* encodes a subunit of acetyl-CoA carboxylase, which participates in fatty acid synthesis. *clpP* and *accD* are even present in highly reduced plastid genomes^7^,^33^,^41^,^42^, from which genes related to photosynthesis as well as many housekeeping genes have been lost. Therefore, it is sometimes hypothesized that the main reason for the retention of plastid genomes in heterotrophic plants is for the coding potential of these genes and that all genes of the translation machinery (i.e., encoding ribosomal components and tRNAs) are retained for translation of their mRNA (e.g. refs 7,9). Surprisingly, it was reported that the *V. macrocarpon*^3^ and *A. unedo*^4^ plastomes lack functional *clpP* and *accD* genes. Additionally, *Actinidia chinensis*, a plant from Actinidiaceae (order Ericales), a relative of Ericaceae, is reported to have lost *clpP*^51^. Herein, we argue in support of an idea that both *clpP* and *accD* are functional in most of the Ericaceae sequenced to date (and Actinidia may also have functional *clpP*). While investigating a short fragment of *accD* located in the plastid genomes of *H. monotropa*, *M. uniflora*, *P. rotundifolia* and *A. unedo*, we found that it is a part of a long open reading frame (ORF) in all these genomes: 1905 bp in *H. monotropa*, 4383 bp in *M. uniflora*, 3141 bp in *A. unedo* and 2466 bp in *P. rotundifolia*. For comparison, the *accD* gene in most plants is approximately 1500 bp long. The closest matches of this ORF to the NCBI nr protein database using BLASTP are to the plastid *accD* gene from Ericales and other angiosperms. According to BLASTP alignments, two regions in each of these ORFs align to *accD* of photosynthetic Ericales: the first is approximately 600 bp (200 aa) long, and the other is approximately 210 bp (70 aa). No matches were retrieved for the remaining part of these ORFs, which consists of multiple AT-rich repeat blocks (Supplementary Fig. 2). To assess whether this ORF may be intact and encodes a protein possibly performing the functions of *accD*, we tested for selection by a site model using PAML, which allows to evaluate the selective pressure acting on each codon of a gene. The results are presented in Fig. 3a. Among 326 columns in a multiple alignment (with each column corresponding to a codon) with no gaps, 67%, 32%, and 1% of the codons are under negative selection, neutral evolution and positive selection, respectively. According to the likelihood ratio test, the p-value of the hypothesis that this ORF has sites evolving under positive selection is 0.13; thus, we suppose that the sequence evolves primarily through a combination of negative selection and neutral evolution. This result provides circumstantial evidence that *accD* is not a pseudogene in Ericaceae. We did not find a long *accD*-containing ORF in *V. macrocarpon* suggesting that *accD* has indeed been lost from the plastome of this species and that it is presumably functionally replaced by a nuclear-encoded gene. However, an unannotated region of ~2800 bp in length that contains *accD*-like sequence is still present. An alternative explanation is that apparent pseudogenization of *accD* in *V. macrocarpon* plastome is an artefact of the sequencing/assembly that created non-triplet indels within the *accD* ORF. It was sequenced using 454 pyrosequencing technology, which has an increased error rate in homopolymeric regions. Such regions are abundant in the *accD* ORF of other Ericaceae. A more evident example of such artefact is the annotation of *accD* as pseudogene in Gruzdev *et al.*^39^. In their plastome assembly, the *accD* ORF has a frameshift due to 1-bp indel in homopolymeric (6A) region. Notably, the sequence of another isolate of *H. monotropa* from the same authors (not published but available in the GenBank, accession number KU640957) has an intact *accD* ORF, strongly suggesting that the frameshift in the published sequence is an error.

In most plants, *clpP* is split into three exons, though loss of the first intron can be observed in several species^7^,^52^,^53^. We found that this gene is present in *M. uniflora*, *H. monotropa*, *V. macrocarpon*, and *P. rotundifolia* and *A. chinensis* (from which it was also thought to be lost), but in an intronless form of a single long exon arising from the joining of the three typical exons (intronless *clpP* is also found in several parasitic Orobanchaceae^33^). Its divergent sequence is most likely the reason why this gene was previously thought to be a pseudogene. Site model analysis using PAML suggested that 37%, 57% and 6% of codons are under negative selection, neutral evolution and positive selection, respectively. According to a likelihood ratio test, the p-value of the hypothesis that this gene has codons under positive selection is 0.011. Sites under negative and positive selection and those evolving neutrally are evenly distributed along the length of the gene (Fig. 3b).

However, the results of the site-wise selection analysis for *clpP* and *accD* should be treated with caution. First, these genes are highly divergent in Ericaceae (Supplementary Table 4), and multiple alignment of divergent sequences may contain alignment mistakes that distort selection analysis^54^. Although a study^54^ indicates that alignments by PRANK result in the most precise estimates of selective pressure, we also tested other multiple alignment tools. They produced similar estimates of proportion of sites under negative selection, positive selection and neutral evolution, although the significance of the presence of positive selection varies (Supplementary Table 5) Second, the number of sequences (four for *accD* and five for *clpP*) is the minimum that can be used in the site model. Thus sampling of additional sequences from Ericaceae is necessary to ascertain the mode of selection of *accD* and *clpP* this family.

Search within the *H. monotropa* transcriptome (unpublished data) demonstrates that *clpP* and *accD* are expressed (supplementary note 1). Interestingly, in *H. monotropa* these genes are situated close to each other and, probably, are expressed as a single polycistronic RNA. Although it is split in the transcriptome assembly into two contigs with lengths 3577 bp and 1230 bp, with the break situated in the middle of *accD*, a continuity of RNA-seq read coverage in the corresponding plastome region and a similarity of coverage between these two transcripts (supplementary note 1) suggests that this is a misassembly, and, in fact, the transcript is single. Regarding *M. uniflora*, transcriptome data are available for BLAST searching within “1000 Plant Genomes Project”^55^ ( https://www.bioinfodata.org/Blast4OneKP/), showing that *clpP* and *accD* are also expressed. These results also support the results of our computational analysis that *clpP* and *accD* are intact.

**Figure 4:**
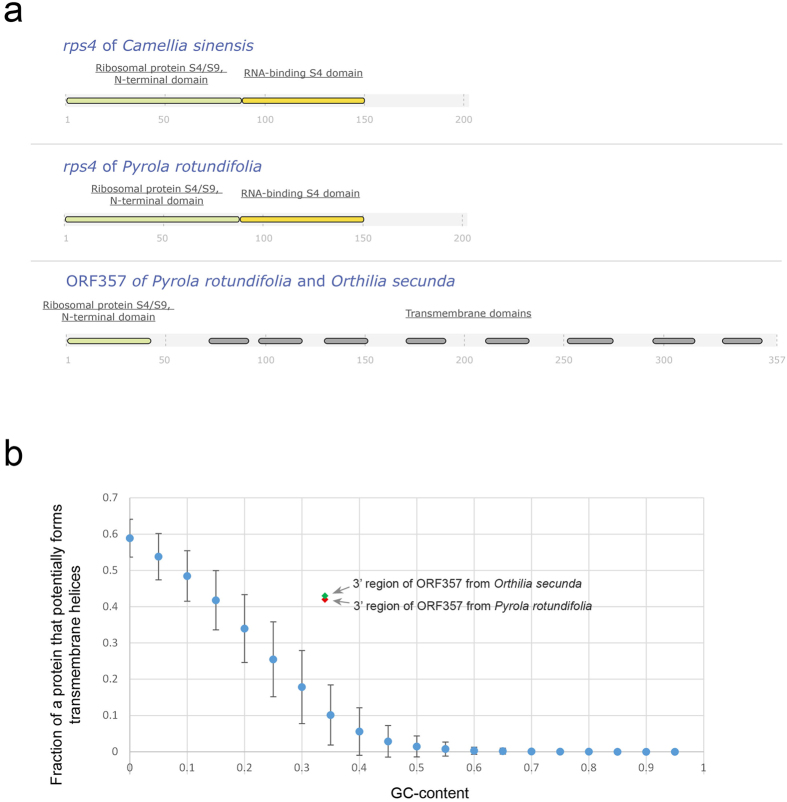
(**a**) Domain structure of the hypothetical product of ORF357; (**b**) transmembrane domain prediction for ORF357 and random ORFs with different GC contents. Blue dots denote mean values of parts of those random ORFs that are predicted to form transmembrane helices by TMHMM, whiskers represent the standard deviation.

Another unusual hypothetical gene is an *rps4*-like gene found in the *P. rotundifolia* plastome. The *rps4* gene encodes the ribosomal protein S4, one of the components of the plastid ribosome, which is present in all sequenced plastid genomes, including the most reduced^42^. The gene length is approximately 600 bp. In addition to a normal, full-length copy of *rps4* in the *P. rotundifolia* plastid genome, a short fragment (114 bp) corresponding to the *rps4* 5′ region is also present. This fragment is the 5′ end of a long ORF of 1074 bp (herein, it will be referred to as ORF357, as it potentially encodes a 357 amino acid polypeptide). A search of similar sequences in the draft assembly of the plastid genome of *Orthilia secunda* (unpublished data, GenBank accession number KU588419), a close relative of *P. rotundifolia*, revealed the presence of this ORF (sequence similarity of 93%). BLAST alignment of the 3′ ends of this ORF from both species to GenBank protein and nucleotide databases yielded no significant matches at e-value <1. Thus, we suggest that the 3′ end is not homologous to any gene and has arisen from an intergenic spacer. Domain analysis by InterPro with TMHMM embedded for predicting transmembrane domains suggests that this 3′ region contains 6 transmembrane domains (Fig. 4a). To evaluate whether the presence of transmembrane domains could be an artefact of domain prediction, we performed computational simulations, generating random ORFs with different GC-contents and estimating by TMHMM software the fraction of amino acids in these ORFs that form transmembrane domains. The result for ORF357 was higher than predicted for a random sequence with the same GC-content (Fig. 4b), lying above the 99th percentile of the randomly generated ORFs both for *P. rotundifolia* and *O. secunda*. Furthermore, according to the BLASTN self-alignment of ORF357, these transmembrane domains are not similar to each other. Thus, we suggest that the domains did not arise via tandem duplications of a single ancestral sequence or at least this duplication was so ancient, that traces of similarity no longer exist. RT-PCR analysis indicated that ORF357 is expressed in *P. rotundifolia* (Supplementary Fig. 3), which supports the hypothesis that this ORF is functional. To explore the mode of selection acting on ORF357, we estimated the ratio of non-synonymous to synonymous substitutions. Pairwise analysis between ORF357 from *P. rotundifolia* and *O. secunda* revealed a dN/dS of 0.83 ± 0.23 for the entire sequence (hereafter, a value after ± denotes the standard error), 0.53 ± 0.48 for the *rps4*-like 5′ end and 0.89 ± 0.25 for the putative transmembrane 3′ end. To test whether the values of dN/dS are significantly different from 1, we performed likelihood ratio tests, which showed that in all three cases the difference is insignificant under significance criterion of p-value less than 0.05. Although dN/dS values close to 1 are considered to be a sign of neutral evolution, they are often a combination of negative selection acting on some sites and positive selection acting on others. As ORF357 sequences are only currently available for two species, it is not yet possible to estimate selection acting on specific codons. We expect that this ORF is present in other species of tribe Pyrolae (the group of Ericaceae to which *P. rotundifolia* and *O. secunda* belong); if so, sequencing will allow for a detailed conclusion regarding the selection acting on this new gene.

## Conclusions

We characterized the plastid genomes of three species of Ericaceae: non-photosynthetic *M. uniflora* and *H. monotropa* and their photosynthetic relative *P. rotundifolia*. Comparative analysis of an expanded set of Ericaceae plastomes allowed to re-examine the gene content and to assume that *clpP* and *accD* that were supposed to be pseudogenes in this family, are functional. Plastomes of non-photosynthetic Ericaceae exhibit extensive gene loss, rearrangements, nucleotide and amino acid biases, a high substitution rate and genes in unusual forms. These traits are a combination of features that are linked with the heterotrophy (loss of photosynthesis-related genes) and features typical for Ericaceae (extensive rearrangements, high repeat content, nucleotide composition bias, transformation of several genes).

With such unusual structure of plastid genomes, Ericaceae are a valuable model for the study of plastome evolution, and furthermore, may provide insight into an intriguing question about the nuclear determinants of plastome structure and coevolution of nuclear and plastid genomes. Now the data on this subject begins to accumulate; e.g. recent study by Zhang *et al.*^56^ that suggests the role of DNA recombination, replication and repair systems in creating plastome complexity. This study was focused on Geraniaceae, the group which is not closely related to Ericaceae, but has many convergences in plastid genome structure (rearrangements, high repeat content, pseudogenes). We expect that characterization and analysis of more plastid genomes and nuclear genes from Ericaceae will allow to test, expand and complement these findings.

## Materials and Methods

### DNA extraction, library preparation, sequencing

DNA was extracted using the CTAB method^57^. For each species, two types of libraries, standard shotgun and long-insert (mate pair), were prepared. The shotgun libraries were prepared using the TruSeq DNA sample prep kit and the mate pair library using the Nextera Mate pair sample prep kit (Illumina) following the manufacturer’s instructions for agarose gel based size selection (Supplementary Table 6). The libraries were sequenced using HiSeq2000 and Miseq instruments (see Supplementary Table 6 for sequencing parameters and output).

### RNA manipulations

RNA was extracted using the RNEasy mini kit (Qiagen) with the addition of the Plant RNA Isolation Aid solution (Ambion) to the lysis buffer. RNA was treated twice with RNase-free DNAse (Qiagen). cDNA synthesis was performed using the MMLV reverse transcription kit (Evrogen). Following reverse transcription, PCR was performed using primers Pyrrot-rps4-F (5′-GGGAGCTTTACCAGGACTAAC-3′) and Pyrrot-rps4-div-R (5′-AATGTTAGTGGACGGTGGTATC-3′) for *rps4*-like ORF357. As a positive control, we also amplified two photosynthesis-related genes – *petB* (Pyrrot-petB-F 5′-ATGAGTAAAGTCTACGATTGGTTC-3′ and Pyrrot-petB-R 5′-AAACGAGTCAAAGTGGATTGTC-3′) and *psaB* (Pyrrot-psaB-F-5′-ACTCGTCGTATTTGGTTTGGGATT-3′ and Pyrrot-psaB-R 5′-TTATTCCATCGGACAAACTCTCC-3′). A negative control with no reverse transcriptase added was also included.

### Preprocessing of reads

Prior to *de novo* assembly, junction adapters were removed from the mate-pairs reads, and the reads were classified into true mate-pairs and paired-end reads using NextClip 0.8^58^, the latter were discarded. Low-quality regions and sequencing adapters were removed from all reads with Trimmomatic 0.32^59^. The long reads produced by MiSeq were trimmed from the 3′ and 5′ ends using a minimum quality of 20 and trimmed from the 3′ end by a sliding window of size 5 with a minimum average quality of 20. Reads with an average quality lower than 20 were discarded. The shotgun libraries reads produced by HiSeq were trimmed to remove bases with a quality lower than 3 from the 3′ end and reads with an average quality lower than 20 were discarded. Reads shorter than 30 bases were discarded. We also used Kmernator 1.2 ( https://github.com/JGIBioinformatics/Kmernator) to remove all k-mers (of length 21) with coverage less than 5.

### Plastid genome assembly

Assembly was performed using SPAdes 3.5^60^, with default parameters, which performs preliminary error correction and assembly with k-mers with lengths 21, 33, 55, 77, 99, 127 (the latter only when long MiSeq reads are used in assembly), with the addition of the ‘careful’ parameter. Identification of contigs of plastid origin was performed by BLASTN 2.2.29+ and TBLASTX 2.2.29+^61^ searches of protein-coding genes and proteins from the plastid genome of *Arbutus unedo* (GenBank accession JQ067650), *Vaccinium macrocarpon* (GenBank accession NC_019616) and *Camellia sinensis* (GenBank accession NC_020019) with a maximum e-value of 10^−5^. Contigs corresponding to plastid genomes were checked for misassembly using REAPR 1.0.17^62^. Scaffolding was performed in the following way. First, we mapped mate-pair reads using CLC Assembly Cell 4.2 ( www.clcbio.com) by requiring at least 80% of the read length to map with identity of at least 99%. We did not use paired-end reads for scaffolding, because due to their short insert sizes they are less capable of resolving repeats. We then visualized mate pair links between the contigs using Circos 0.67^63^. Additionally, to identify possibly overlapping sequences between the ends of the contigs, we performed a BLASTN alignment of the plastid contig set to itself. The result was inspected visually by plot construction in Circos. To assess for misassembled repeat regions, we mapped all reads by CLC Assembly Cell to the contigs and visually searched for areas of increased coverage. Combined analyses of mate-pair links and repeats allowed us to determine the order of the contigs in the plastid genome. We manually joined the contigs into a scaffold and filled the gaps using GapFiller 1.10^64^.

It is difficult to precisely assemble regions abundant in repeats, which are present in the *P. rotundifolia* and *M. uniflora* plastomes. Thus, we performed three steps to assemble them as strictly as possible:

(1) We mapped mate-pair reads to the region containing the repeats.

(2) We calculated an average insert size of mate-pairs in which the left read is located to the left of the repeat-containing region and the right read to the right of this region.

(3) If the average insert length is shorter than the average insert length at regions of a genome that do not contain repeats, then the number of copies of tandem repeats is underestimated; however, if it is larger, then the number of copies of tandem repeats is overestimated. In the first case, we added one copy of a tandem repeat unit, and in the second case, we removed one. These procedures were iteratively performed until the average insert size in the tandem repeat region became equal to the average insert size in non-repeat regions.

The copies of repeats in a tandem repeat regions can be divergent. To account for this, we mapped mate-pair reads by CLC Assembly Cell, demanding that 80% of a reads’ length map with an identity of at least 98%; we then replaced nucleotides at positions in which a consensus of mapped reads suggested another nucleotide than that present in a current version of the sequence. Then, we remapped the reads. This operation was iteratively performed until no discrepancies between the mapping and the contig sequence remained.

To verify the assembled plastid genomes, we mapped all reads by CLC Assembly Cell, demanding that at least 80% of the read length map with an identity of at least 99% and ensuring no sites with decreased coverage. Additionally, we mapped the mate-pair reads and estimated the average insert size over all positions of the genomes and a number of mate-pair inserts (“coverage by inserts”) that span each genome position (see Supplementary Fig. 4). The uniformity of insert size distribution suggests no unnoticed artificially made insertions and deletions in the sequence, and the absence of sharp declines in the “coverage by inserts” indicate no incorrect contig connections.

### Annotation

The initial annotation was performed using the on-line service DOGMA^65^. The annotation was performed with relaxed parameters: an e-value < 10^−5^ and a sequence similarity to reference genes >25%. The annotations were then checked manually. To verify borders of intronless genes, we calculated ORFs in the plastid genomes using CLC Genomics Workbench 7.0 by considering sequences that start with any of 7 possible plastid start codons (ATG, TTG, GTG, CTG, ATA, ATT, ATC), end with a stop codon, have no internal stop codons and are longer than 90 bp. To refine the predictions of tRNAs, we scanned the entire plastid genomes with an online version of tRNAscan-SE 1.2.11^66^ at http://selab.janelia.org/tRNAscan-SE/ using both the general and organellar tRNA modes of the program. Additionally, we verified the gene prediction made by DOGMA by generating BLASTN and TBLASTN alignments of the genes and proteins of *Camellia sinensis* (as one of the most studied plants in Ericales) with a maximum e-value of 10^−5^ and by inspecting the alignments visually. Verification of borders of genes with introns was made similarly, by aligning genes that have introns in *C. sinensis* to assembled genomes by BLASTN and TBLASTN, but more sensitively - with a maximum e-value of 10, and word sizes of 7 for BLASTN and 2 for TBLASTN. Finally, we performed a multiple alignment of our three plastid genomes using mVista^67^ to ascertain the presence of any unusually conserved regions in the genomes, regions that could correspond to unnoticed genes. The maps of the plastid genomes (Fig. 1) were built using the on-line tool OGDRAW^68^. Plastome sequences are deposited in NCBI Genbank under accession numbers KU878156 (*Hypopitys monotropa*), KX228067 (*Monotropa uniflora*) and KU833271 (*Pyrola rotundifolia*).

### Analysis of the genomes

The domain structures of ORF357, *accD* and *clpP* were analysed using the InterPro server^69^ at http://www.ebi.ac.uk/interpro/.

A computational simulation was performed to evaluate whether the transmembrane domains of ORF357 are an artefact of domain prediction. Utilizing random sequences of 999 bp each, we removed stop codons from the respective 333 codons and translated the remaining codons into an amino acid sequence. Potential transmembrane helices in this randomly generated amino acid sequence were predicted by TMHMM 2.0^70^. This operation was performed for sequences with all possible GC-contents from 0 to 1 with a step of 0.05. For each GC-content, 1000 random sequences were generated, and then we calculated for each GC-content the average fraction of sequence length that potentially forms a transmembrane helix and a standard deviation of this value. To compare individually the percent of sequence of ORF357 potentially forming transmembrane helices with that parameter of random ORFs, we also generated 1000 random ORFs with GC-contents as in ORF357 of *Pyrola* and as in *Orthilia* and calculated the percentile in the random ORFs above which that value falls.

Repeats in the genomes were tabulated using BLASTN alignment of the genomes to themselves with a word size of 9 bp. For Fig. 1, we compiled all repeats with a minimum length of 20 bp and a minimum sequence similarity of 95%, irrespective of the e-value. Circos was then used to generate diagrams of the repeats. The same set of parameters was used to calculate repeats for Supplementary Table 1. For dot plot diagrams (Supplementary Figs 1 and 2), BLASTN self-alignment in NCBI Genome Workbench 2.9.5 ( http://www.ncbi.nlm.nih.gov/tools/gbench/ was performed with a word size of 9 bp and a maximum e-value of 10^−5^. The low-complexity filter (“-dust no” option) was switched off for all BLAST alignments.

To build a phylogenetic tree (Fig. 2), we used sequences of plastid protein-coding genes, plastid rDNAs, nuclear 18s rDNAs and the nuclear internal transcribed spacer (ITS) of *M. uniflora*, *H. monotropa*, *A. unedo*, *V. macrocarpon*, *P. rotundifolia* and *C. sinensis*. Nuclear 18S rDNA and the ITS sequences for our species were found among the contigs from the *de novo* assembly by BLASTN alignment of the respective sequences from close relatives deposited in GenBank. We verified the found sequences by making their BLASTN alignment to NCBI NT database. We used consensus sequences, generated by SPAdes, and hence did not account for possible polymorphisms between genomic copies of the sequences. The 5.8S rDNA situated between ITS1 and ITS2 was removed. Multiple alignments of the sequences from these four sets were then performed separately. The common protein-coding genes of the aforementioned species were aligned using a combination of Muscle^71^ and TranslatorX^72^ with default options, which allows alignment of protein-coding genes by taking into account a respective amino acid alignment. Poorly aligned regions were removed using Gblocks^73^ in the codon mode with the most liberal settings for poorly aligned region removal available on the Gblocks server ( http://molevol.cmima.csic.es/castresana/Gblocks_server.html). The plastid rDNA, nuclear 18S rDNA and ITS sequences were aligned separately using Muscle, and poorly aligned regions were removed by Gblocks in a nucleotide mode, also with the most liberal settings. The four respective alignments were concatenated, and a phylogenetic tree was built using RAxML 8.1.20^74^. We used the GTR+Gamma model and 5 partitions, with RAxML calculating its own parameters of the GTR+Gamma model for each. The partitions were as follows: (1) the first and second bases in each codon of protein-coding genes; (2) the third base in each codon of protein-coding genes (they are often synonymous and, therefore, accumulate mutations faster); (3) plastid rDNAs; (4) nuclear 18S rDNAs; (5) nuclear ITS. RAxML builds 100 starting trees, and after topology estimation, it performs 1000 iterations of fast bootstrap analysis.

To estimate the selective pressure acting on protein-coding plastid genes, a multiple alignment of the concatenated sequences of those genes was performed using a combination of Muscle and TranslatorX with default parameters on TranslatorX server. The dN/dS (the ratio of rates of non-synonymous and synonymous substitutions) was then calculated using the branch model in PAML 4.7^75^ with the topology estimated as indicated above. Statistical significance of difference between substitution accumulation rates of the species was estimated by Tajima’s relative rate test in MEGA 6^76^. We performed pairwise comparisons between all pairs of the five studied Ericaceae (ten pairwise comparisons), using *Camellia* as an outgroup. To calculate pairwise dN/dS values between the sequences of ORF357 from *Pyrola* and *Orthilia*, we aligned them using Muscle and TranslatorX and then calculated dN/dS in PAML in the pairwise mode. To test whether these values are significantly different from 1, we performed the same computations, while fixing dN/dS to 1 and then comparing likelihoods between the models with fixed and floating dN/dS by the likelihood ratio test.To estimate the selective pressure acting on the genes *clpP* and *accD* in Ericaceae, we generated multiple alignments of these sequences using the webPRANK server^77^ ( http://www.ebi.ac.uk/goldman-srv/webprank/) in the codon alignment mode with default parameters. For the analyses of both *clpP* and *accD* we used all species of Ericaceae where they are intact, i.e. *Uniflora*, *Hypopitys*, *Vaccinium* and *Pyrola* for *clpP*, and *Uniflora*, *Hypopitys*, *Arbutus* and *Pyrola* for *accD*. We chose PRANK for alignments of *clpP* and *accD* because it has been noted that for highly divergent sequences, dN/dS analysis performed on alignments generated by PRANK provides more precise results than other alignment tools^54^. Then, we ran PAML in site models M1a (which allows for negative selection and neutral evolution) and M2a (which allows for negative selection, neutral evolution and positive selection). To calculate the p-value of the hypothesis that positive selection exists, a likelihood ratio test was performed for the likelihoods of these two models. In all calculations performed by PAML, the Gy94_3 × 4codon model was used, and the transition/transversion ratio was estimated by PAML with a starting value of 2.0. As a model for codon substitutions we used the “basic” model, which is a simplified version of the model of Goldman and Yang^78^. In the alignments, the columns with gaps were not analysed (cleandata = 1 option). To estimate the effect of the alignment tool on selection estimation, we also performed multiple alignments of *accD* and *clpP* genes by combinations of TranslatorX and, separately, PRANK, MUSCLE, MAFFT^79^, ClustalW^80^ and T-Coffee^81^. The alignments were made on the TranslatorX server with default parameters.

To calculate the percentage of identity among protein-coding genes and proteins, we aligned them using Muscle+TranslatorX and Muscle, respectively (in both cases with default parameters) and calculated the percent identity in BioEdit 7.2.5^82^. Alignments generated by PRANK usually contain excessive amounts of short gaps, which distort the results of sequence identity calculations. The codon usage analysis was performed in CodonW 1.4.2^83^.

## Additional Information

**How to cite this article**: Logacheva, M. D. *et al.* Comparative analysis of plastid genomes of non-photosynthetic Ericaceae and their photosynthetic relatives. *Sci. Rep.* **6**, 30042; doi: 10.1038/srep30042 (2016).

## Supplementary Material

Supplementary Information

## Figures and Tables

**Table 1:** General characteristics of Ericaceae plastomes.

Species	Plastome length, bp	GC content, %	Total number of genes*	Number of protein-coding genes*	Number of rRNA-coding genes*	Number of tRNA-coding genes*
*Arbutus unedo*	150 897	37.3	109	75	4	30
*Vaccinium macrocarpon*	176 045	36.8	100	68	4	28
*Pyrola rotundifolia*	168 995	35.7	101	67	4	30
*Monotropa uniflora*	45 111	28.9	40	22	4	14
*Hypopitys monotropa*	35 062	34.4	44	22	4	18

^*^–duplicated genes are counted as a single gene. Pseudogenes are not counted.

**Table 2:** Gene content in Ericaceae plastomes.

Species	*Arbutus unedo*	*Vaccinium macrocarpon*	*Pyrola rotundifolia*	*Monotropa uniflora*	*Hypopitys monotropa*
Ribosomal proteins, small subunit	rps2, rps3, rps4, **rps7**, rps8, rps11, rps12*, rps14, **rps15**, Ψrps16, rps18, rps19	rps2, rps3, rps4, rps7, rps8, rps11, Ψrps12, rps14, **rps15**, **Ψrps16**, rps18, rps19	rps2, rps3, rps4, rps4-like gene (ORF357), **rps7**, rps8, rps11, rps12*, rps14, rps15, rps16, rps18, rps19	rps2, rps3, rps4*, rps7, rps8, rps11, rps12, **rps14**, rps18, rps19	rps2, rps3, rps4, rps7, rps8, rps11, rps12, rps14, rps18, Ψrps19
Ribosomal proteins, large subunit	rpl2, rpl14, rpl16, rpl20, rpl22, rpl23, rpl32, rpl33, rpl36	rpl2, rpl14, rpl16, rpl22*, rpl23, rpl32, rpl33, rpl36	Ψrpl2, rpl14, rpl16, rpl20, rpl22, rpl23, **rpl32**, rpl33, rpl36	rpl2, rpl14, rpl16, rpl20, rpl22, rpl33, rpl36	rpl2, rpl14, rpl16, rpl20, rpl22, rpl23, rpl32, rpl33, rpl36
Ribosomal RNA	**rrn16**, **rrn23**, **rrn5**, **rrn4.5**	**rrn16**, **rrn23**, **rrn5**, **rrn4.5**	**rrn16**, **rrn23**, **rrn5**, **rrn4.5**	**rrn16**, rrn23, rrn5, rrn4.5	rrn16, rrn23, rrn5, rrn4.5
Transfer RNA	**trnA-UGC**, trnC-GCA, trnD-GUC, trnE-UUC, trnF-GAA, trnG-GCC, trnG-UCC, **trnH-GUG**, **trnI-CAU**, **trnI-GAU**, trnK-UUU, **trnL-CAA**, trnL-UAA, **trnL-UAG**, trnM-CAU, trnfM-CAU, **trnN-GUU**, trnP-UGG, trnQ-UUG, **trnR-ACG**, trnR-UCU, trnS-GCU, trnS-GGA, trnS-UGA, trnT-GGU, trnT-UGU, **trnV-GAC**, trnV-UAC, trnW-CCA, trnY-GUA	**trnA-UGC**, trnC-GCA, trnD-GUC, trnE-UUC, trnF-GAA, trnG-UCC, **trnH-GUG**, trnI-CAU, **trnI-GAU**, trnL-CAA, trnL-UAA, **trnL-UAG**, trnM-CAU, trnfM-CAU, **trnN-GUU**, trnP-UGG, trnQ-UUG, **trnR-ACG**, trnR-UCU, trnS-GCU, trnS-GGA, trnS-UGA, trnT-GGU, trnT-UGU, trnV-GAC, trnV-UAC, trnW-CCA, trnY-GUA	**trnA-UGC**, trnC-GCA, trnD-GUC, trnE-UUC, trnF-GAA, trnfM-CAU, trnG-GCC, trnG-UCC, **trnH-GUG**, **trnI-CAU**, **trnI-GAU**, trnK-UUU, **trnL-CAA**, trnL-UAA, **trnL-UAG**, trnM-CAU, **trnN-GUU**, trnP-UGG, trnQ-UUG, **trnR-ACG**, trnR-UCU, trnS-GCU, trnS-GGA, trnS-UGA, trnT-GGU, trnT-UGU, **trnV-GAC**, trnV-UAC, trnW-CCA, trnY-GUA	trnC-GCA, trnD-GUC, trnE-UUC, trnF-GAA, trnH-GUG, trnI-CAU, trnfM-CAU, **ΨtrnM-CAU**, trnN-GUU, trnP-UGG, trnQ-UUG, trnS-GCU, **trnS-UGA**, **trnT-GGU**, ΨtrnV-GAC*, trnW-CCA, trnY-GUA	trnC-GCA, trnD-GUC, trnE-UUC, trnF-GAA, trnH-GUG, trnI-CAU, trnfM-CAU, trnL-UAG, trnM-CAU, trnN-GUU, trnP-UGG, trnQ-UUG, trnR-ACG, trnR-UCU, trnS-UGA, trnS-GCU, trnW-CCA, trnY-GUA
Photosystem I	psaA, psaB, **psaC**, psaI, psaJ	psaA, psaB, **psaC**, psaI, psaJ	psaA, psaB, psaC, psaI, psaJ		ΨpsaB
Photosystem II	psbA, psbB, psbC, psbD, psbE, psbF, psbH, psbI, psbJ, psbK, psbL, psbM, psbN, psbT, psbZ	psbA*, psbB, psbC, psbD, psbE, psbF, psbH, psbI, psbJ, psbK, psbL, psbM, psbN, psbT, psbZ	psbA, psbB, psbC, psbD, psbE, psbF, psbH, psbI, psbJ, psbK, psbL, psbM, psbN, psbT, psbZ		
Cytochrome b6/f complex	petA, petB, petD, petG, petL, petN	petA, petB, petD, petG, petL, petN	petA, petB, ΨpetD, petG, petL, petN		
Photosynthesis – others	rbcL, **ccsA**, ycf3, ycf4	rbcL, **ΨccsA**, ycf3, ycf4	rbcL, ccsA*, ycf3, ycf4		ΨccsA
NADH-dehydrogenase	**ndhA**, **ndhB**, ndhC, **ndhD**, **ndhE**, ndhF, **ndhG**, **ndhH**, **ndhI**, ndhJ, ndhK	**ndhA**, ndhB, ndhC, **ndhD**, **ndhE**, ndhF, **ΨndhG**, **ndhH**, **ndhI**, ndhJ, ΨndhK	ΨndhA, **ndhB**, ΨndhC, ΨndhD, ΨndhE, ΨndhF, ndhG, ΨndhH, ΨndhI, ΨndhJ, ΨndhK		ΨndhB
RNA polymerase	rpoA, rpoB, rpoC1, rpoC2	rpoA, rpoB, rpoC1, rpoC2	rpoA, rpoB, rpoC1, rpoC2		
ATP synthase	atpA, atpB, atpE, atpF, atpH, atpI	atpA, atpB, atpE, ΨatpF, atpH, atpI	atpA, atpB, atpE, atpF, atpH, atpI		ΨatpB
Others	matK, infA, cemA, accD, ΨclpP, Ψycf1	matK, ΨinfA, cemA, ΨaccD, clpP, Ψycf2	matK, infA, cemA, accD, clpP, **Ψycf2**	matK, infA, accD, **clpP**	matK, infA, accD, clpP

The names of genes situated within inverted repeats are in bold. Genes with copies both in IR and single-copy regions or that are situated partially in the IR and partially in single-copy regions are marked with an asterisk. Pseudogenes are marked with Ψ. If both the normal and pseudogenized copy of a gene are present in the genome, only the normal one is indicated here.

**Table 3:** GC content in Ericaceae and *Camellia sinensis*.

Species	GC1	GC2	GC3	Total GC
*H. monotropa*	0.424	0.377	0.263	0.355
*M. uniflora*	0.365	0.332	0.171	0.289
*P. rotundifolia*	0.438	0.399	0.248	0.362
*V. macrocarpon*	0.447	0.4	0.266	0.371
*A. unedo*	0.459	0.409	0.273	0.38
*C. sinensis*	0.461	0.406	0.268	0.378

GC1, GC2 and GC3 refer to the first, second and third positions of codons in protein-coding genes.
