# Supplementary material for: Comparative analysis of plastid genomes of non-photosynthetic Ericaceae and their photosynthetic relatives
Source: Sci Rep. 2016 Jul 25;6:30042. doi: 10.1038/srep30042 (PMC4958920; doi:10.1038/srep30042)
Supplement: Supplementary Information [file srep30042-s1.pdf]

Manuscript title: **Comparative analysis of plastid genomes of non-photosynthetic Ericaceae and their photosynthetic relatives**

Authors: Maria D. Logacheva, Mikhail I. Schelkunov, Viktoria Y. Shtratnikova, Maria V. Matveeva, Aleksey A. Penin

Supplementary figures and tables.

Supplementary figure 1.

Location of repeats in the plastid genomes of Ericaceae and two species from Ericales (*Actinidia chinensis* and *Camellia sinensis*). The dot-plot charts are built based on BLASTN self-alignment of the genomes. Detailed plots for regions that are particularly rich in repeats are shown in blue frames. Genomic positions are subscribed on the axes.

Supplementary figure 2.

Location of repeats within open reading frames containing an accD-like fragment. Dot-plots were built based on BLASTN self-alignments of respective sequences. Axes denote nucleotide positions within the open reading frames.

Supplementary figure 3.

Picture of a gel showing the expression of ORF357 in *Pyrola rotundifolia*. 1 – DNA ladder (0.1 – 1 Kb), 2 – empty, 3 – ORF357, 4 – no reverse transcriptase control, 5 – empty, 6 – petB, 7 – no reverse transcriptase control, 8 – empty, 9 – psaB, 10 – no reverse transcriptase control, 11 – empty, 12 - DNA ladder (0.1 – 1 Kb).

Supplementary figure 4.

Support of plastid genomes structure by mate-pair reads. The X-axis denotes a position in a plastid genome. The left Y-axis and a red line denote coverage by mate-pair inserts, i.e., how many mate-pairs reads there are in which the left read lies to the left of this position and the right one to the right of this position. The right Y-axis and a blue line denote the average insert length of such mate-pair reads over this position.

Supplementary table 1.

Fraction of repeats in Ericaceae plastomes.

Supplementary table 2.

Codon usage in plastid genomes of Ericaceae and *Camellia sinensis*.

Supplementary table 3.

Comparison of substitution rates in Ericaceae.

Supplementary table 4.

Sequence similarity for *accD* and *clpP* in Ericaceae.

Supplementary table 5.

Comparison of effect of an alignment tool on a selective pressure estimate for *accD* and *clpP*.

Supplementary table 6.

Characteristics of libraries, sequencing parameters and output.

Supplementary note 1.

Characteristics of the transcripts of *Hypopitys monotropa* with significant similarity to *accD* and *clpP* and sequences of contigs containing *clpP* and *accD* ORF.

Supplementary figure 1, whole genome dot plots.

Monotropa uniflora

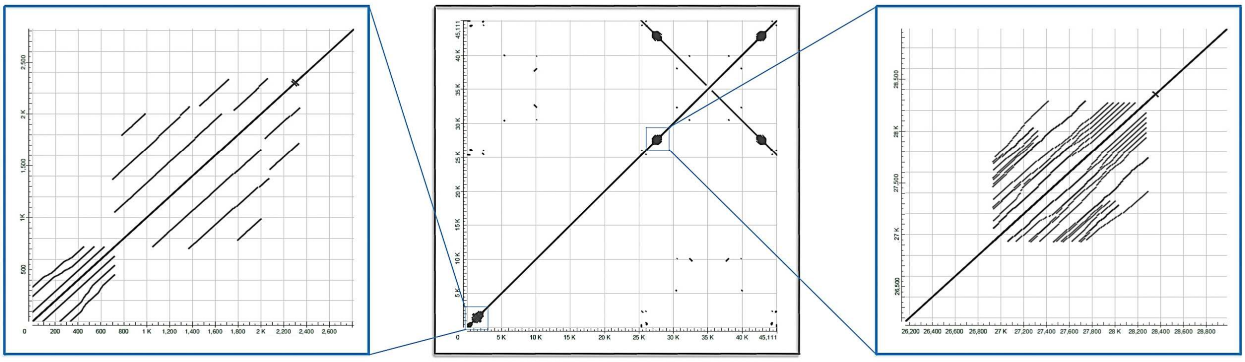

Hypopitys monotropa

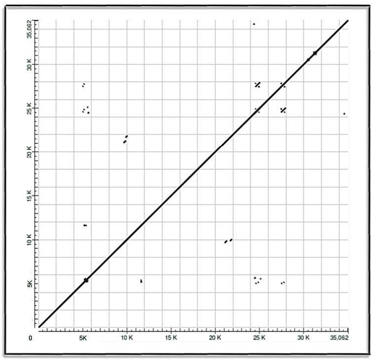

Arbutus unedo

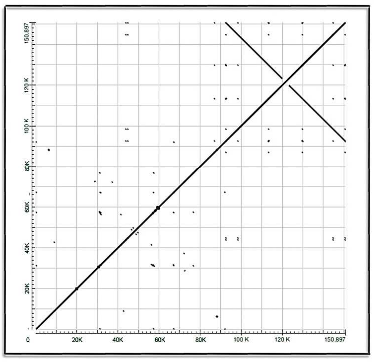

Vaccinium macrocarpon

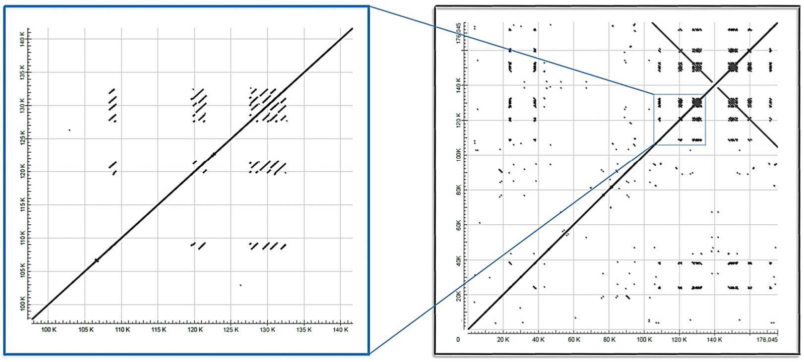

Supplementary figure 1, continued.

Pyrola rotundifollia

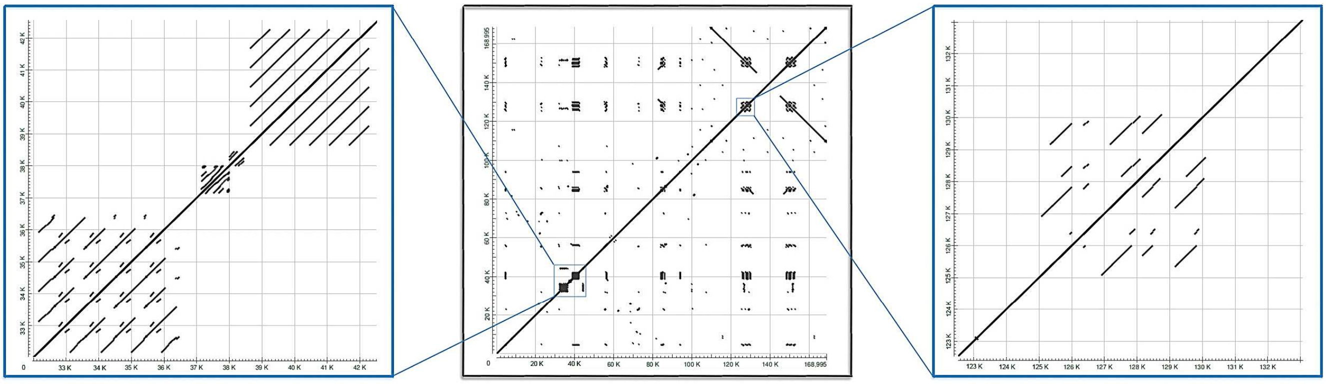

Actinidia chinensis

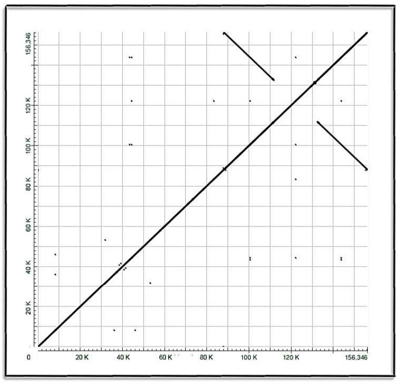

Camellia sinensis

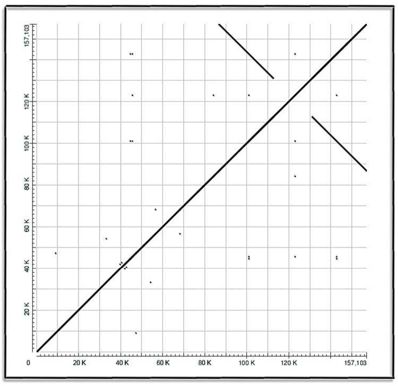

Supplementary figure 2, AccD dot plots.

Monotropa uniflora

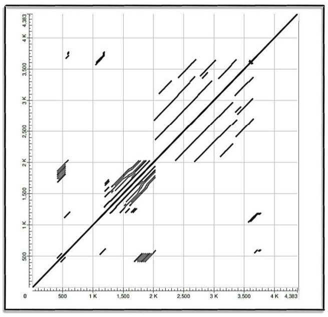

Hypopitys monotropa

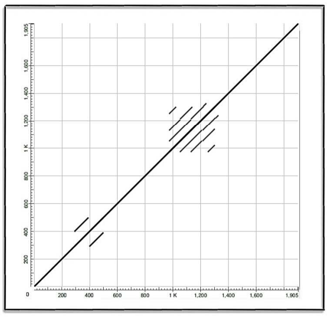

Arbutus unedo

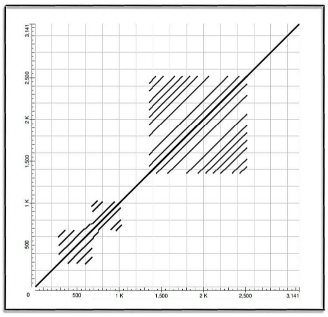

Pyrola rotundifollia

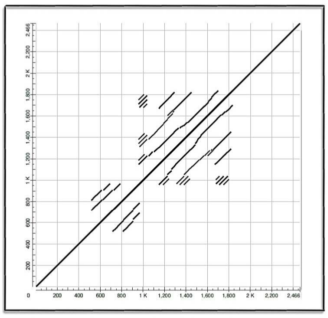

Actinidia chinensis

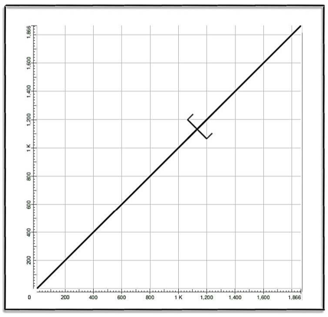

Camellia sinensis

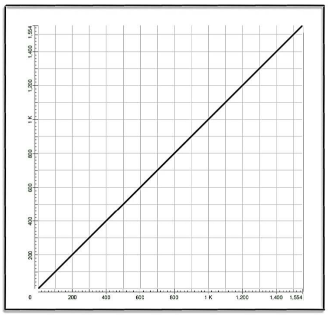

Supplementary figure 3. Picture of a gel showing the expression of ORF357 in Pyrola rotundifolia.
1 – DNA ladder (0.1 – 1 Kb), 2 – empty, 3 – ORF357, 4 – no reverse transcriptase control, 5 – empty, 6 – petB, 7 – no reverse transcriptase control, 8 – empty, 9 – psaB, 10 – no reverse transcriptase control, 11 – empty, 12 - DNA ladder (0.1 – 1 Kb).

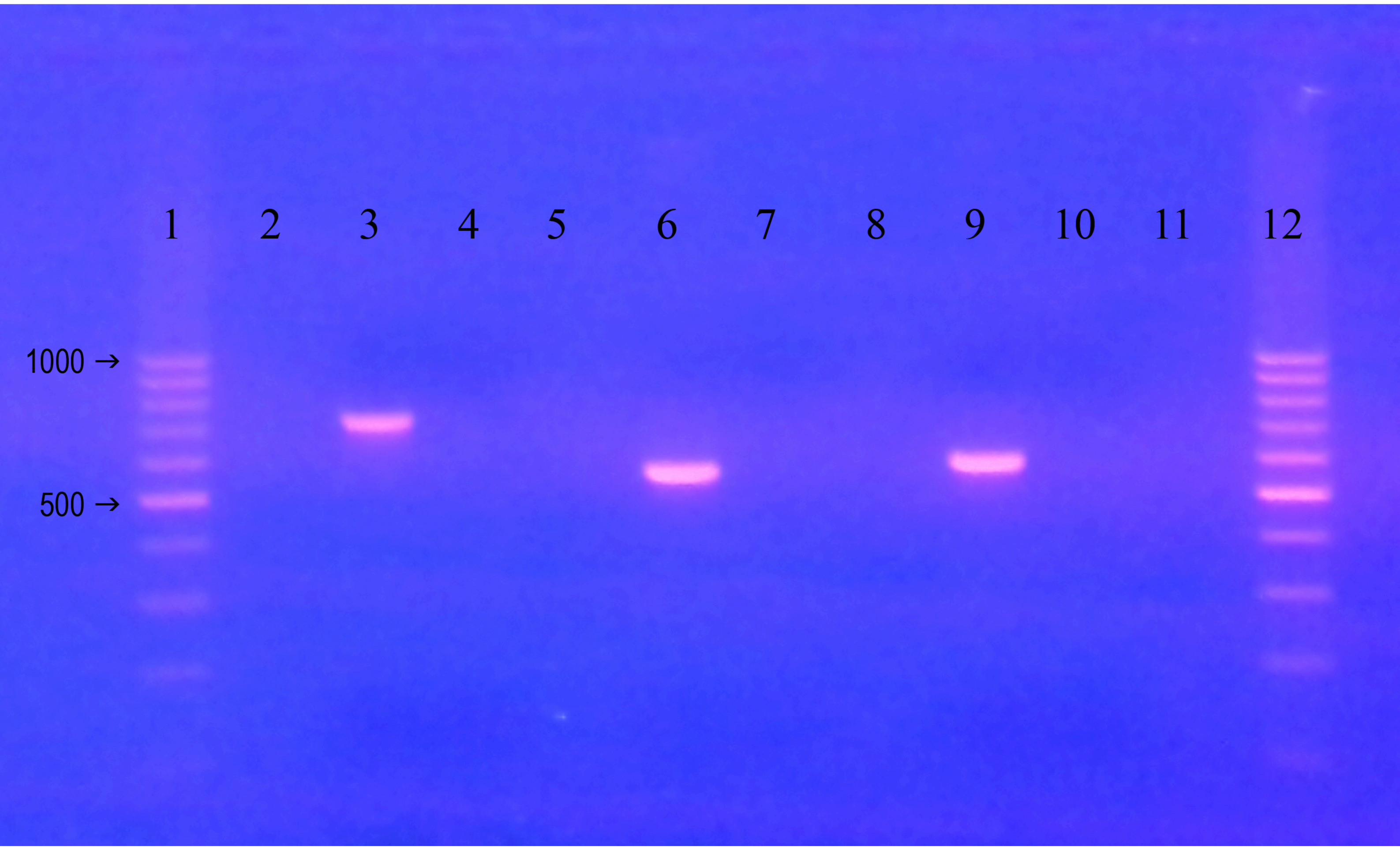

Supplementary figure 4. Support of plastid genomes structure by mate-pairs reads.

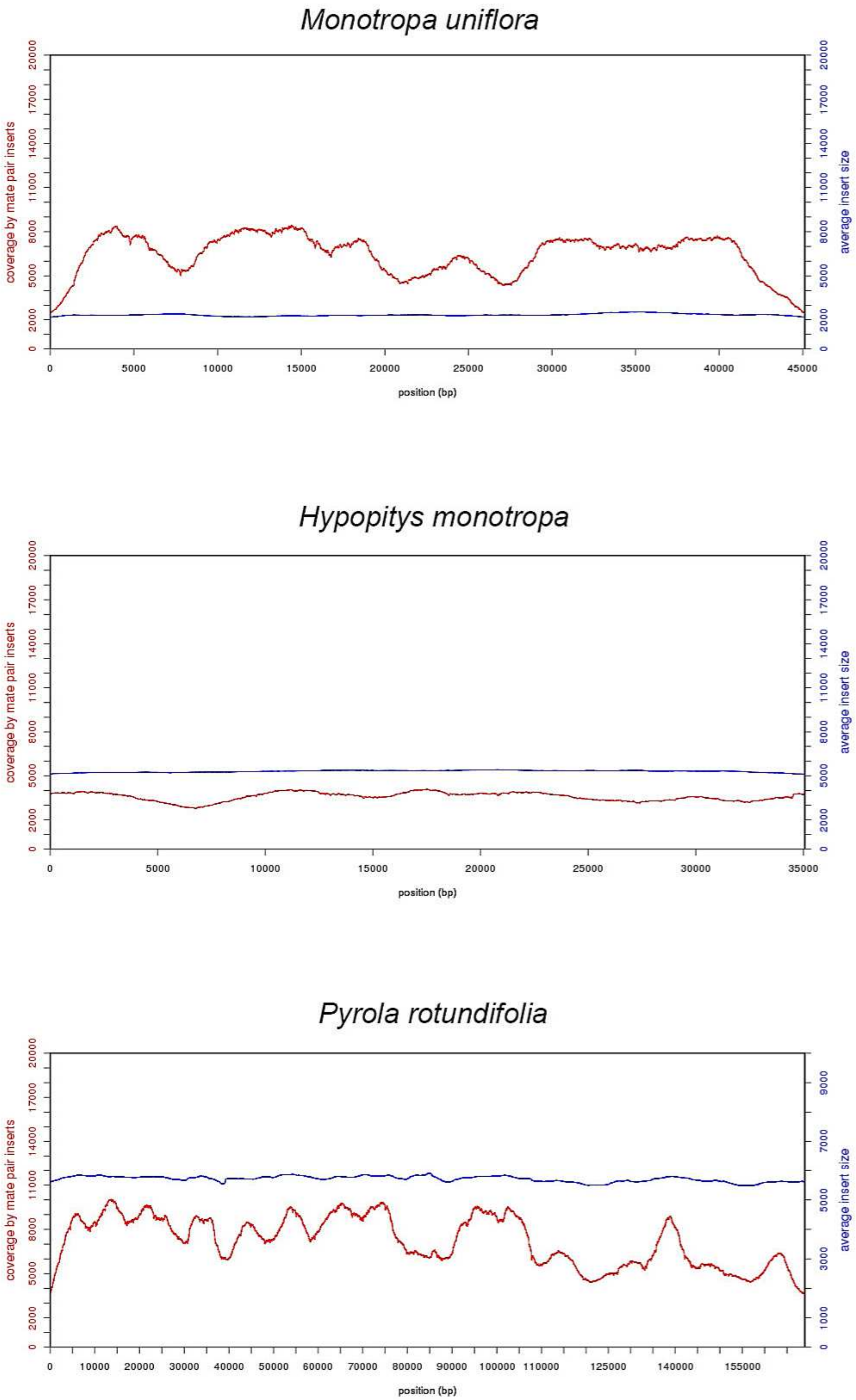

Supplementary table 1. Repeats in plastid genomes of Ericaceae

The table describes amount of repeats of different length in plastid genomes of Ericaceae. Numbers in parentheses denote numbers of such repeats per 1 kbp of genome

Species	*Monotropa uniflora*		*Hypopitys monotropa*		*Pyrola rotundifolia*		*Vaccinium macrocarpon*		*Arbutus unedo*		*Camellia sinensis*		*Arabidopsis thaliana*	
Repeat orientation	direct	reverse	direct	reverse	direct	reverse	direct	reverse	direct	reverse	direct	reverse	direct	reverse
20-50 bp	**21** (0.590)	**20** (0.562)	**13** (0.371)	**6** (0.171)	**61** (0.421)	**54** (0.372)	**50** (0.353)	**51** (0.360)	**16** (0.130)	**20** (0.162)	**24** (0.183)	**21** (0.160)	**51** (0.398)	**49** (0.382)
51-100 bp	**1** (0.028)	**0** (0.000)	**8** (0.228)	**0** (0.000)	**3** (0.021)	**9** (0.062)	**10** (0.071)	**2** (0.014)	**3** (0.024)	**2** (0.016)	**0** (0.000)	**0** (0.000)	**0** (0.000)	**0** (0.000)
101-500 bp	**0** (0.000)	**1** (0.028)	**4** (0.114)	**0** (0.000)	**18** (0.124)	**23** (0.159)	**9** (0.063)	**3** (0.021)	**5** (0.041)	**6** (0.049)	**0** (0.000)	**0** (0.000)	**0** (0.000)	**0** (0.000)
>501 bp	**0** (0.000)	**0** (0.000)	**0** (0.000)	**0** (0.000)	**15** (0.103)	**10** (0.069)	**3** (0.021)	**2** (0.014)	**0** (0.000)	**1** (0.008)	**0** (0.000)	**0** (0.000)	**0** (0.000)	**0** (0.000)

Supplementary table 2 - codon usage in plastid genomes of Ericaceae and Camellia sinensis							
The three numbers denote, respectively, 1.usage of this codon among all codons of this amino acis (in percents) 2.number of such codons per 1000 codons in a genome 3.total number of such codons among all genes of a genome							
		Hypopitys monotropa	*Monotropa uniflora*	*Pyrola rotundifolia*	*Vaccinium macrocarpon*	*Arbutus unedo*	*Camellia sinensis*
Phe	UUU	79 (28, 68)	90 (38, 92)	75 (28, 67)	70 (26, 64)	67 (24, 57)	70 (26, 63)
	UUC	21 (7, 18)	10 (4, 10)	25 (9, 22)	30 (11, 28)	33 (12, 28)	30 (11, 27)
Leu	CUA	11 (10, 25)	12 (12, 29)	13 (12, 29)	15 (14, 34)	14 (13, 31)	13 (11, 27)
	CUG	4 (4, 10)	3 (3, 7)	5 (4, 10)	6 (6, 14)	4 (4, 10)	6 (5, 13)
	UUG	25 (24, 57)	13 (13, 31)	21 (19, 47)	24 (22, 53)	21 (20, 48)	24 (21, 51)
	CUC	5 (5, 12)	4 (4, 9)	6 (6, 14)	4 (3, 8)	5 (5, 12)	5 (5, 11)
	CUU	18 (17, 42)	13 (12, 30)	19 (17, 42)	18 (17, 41)	21 (20, 48)	19 (17, 41)
	UUA	37 (35, 85)	55 (54, 130)	35 (32, 78)	33 (30, 73)	33 (31, 75)	32 (28, 68)
Ile	AUU	49 (46, 111)	51 (62, 149)	49 (45, 109)	48 (41, 99)	49 (41, 100)	46 (43, 103)
	AUA	37 (35, 84)	39 (47, 113)	37 (34, 82)	37 (32, 77)	34 (29, 69)	34 (31, 75)
	AUC	13 (12, 30)	10 (12, 28)	15 (14, 33)	15 (13, 31)	17 (14, 34)	20 (18, 44)
Met	AUG	100 (22, 54)	100 (19, 45)	100 (24, 59)	100 (21, 51)	100 (25, 60)	100 (25, 59)
Val	GUC	11 (5, 13)	4 (2, 4)	15 (7, 17)	13 (7, 17)	18 (10, 23)	9 (5, 12)
	GUU	39 (19, 45)	47 (20, 48)	38 (18, 44)	39 (21, 52)	34 (18, 43)	43 (23, 54)
	GUA	37 (18, 43)	38 (16, 39)	32 (16, 38)	32 (17, 42)	31 (17, 40)	33 (18, 42)
	GUG	13 (6, 15)	12 (5, 12)	15 (7, 18)	17 (9, 22)	17 (9, 22)	15 (8, 19)
Ser	AGC	8 (5, 12)	3 (2, 4)	5 (3, 8)	9 (6, 14)	5 (3, 8)	4 (3, 6)
	UCG	6 (3, 8)	6 (4, 9)	11 (7, 17)	8 (5, 13)	8 (5, 13)	9 (6, 15)
	UCA	21 (12, 30)	29 (18, 43)	20 (13, 31)	19 (12, 29)	18 (11, 27)	20 (13, 32)
	UCU	29 (17, 41)	27 (16, 39)	27 (18, 43)	33 (20, 50)	33 (21, 51)	32 (22, 52)
	UCC	15 (9, 22)	8 (5, 12)	17 (11, 27)	12 (8, 19)	18 (11, 27)	15 (10, 25)
	AGU	20 (12, 29)	27 (17, 40)	20 (13, 32)	18 (11, 28)	18 (12, 28)	20 (13, 32)
Pro	CCU	39 (14, 33)	47 (15, 36)	41 (15, 37)	34 (13, 31)	31 (12, 29)	34 (13, 31)
	CCA	33 (12, 28)	32 (10, 25)	30 (11, 27)	32 (12, 29)	34 (13, 32)	34 (13, 31)
	CCC	19 (7, 16)	14 (5, 11)	20 (7, 18)	24 (9, 22)	24 (9, 22)	22 (8, 20)
	CCG	8 (3, 7)	6 (2, 5)	10 (4, 9)	9 (3, 8)	11 (4, 10)	10 (4, 9)
Thr	ACA	29 (13, 32)	43 (18, 43)	34 (16, 40)	24 (12, 30)	29 (14, 34)	32 (15, 36)
	ACC	25 (12, 28)	9 (4, 9)	20 (9, 23)	24 (12, 30)	24 (12, 28)	22 (10, 24)
	ACG	10 (5, 11)	5 (2, 5)	8 (4, 9)	16 (8, 20)	10 (5, 12)	11 (5, 12)
	ACU	36 (17, 40)	44 (18, 44)	38 (18, 44)	36 (18, 45)	36 (17, 42)	35 (16, 39)
Ala	GCG	10 (5, 12)	6 (3, 7)	8 (4, 10)	9 (5, 12)	9 (5, 12)	8 (5, 11)
	GCC	15 (8, 19)	10 (5, 11)	17 (9, 22)	10 (5, 13)	13 (7, 18)	16 (9, 22)
	GCA	38 (20, 48)	39 (18, 43)	38 (21, 51)	43 (23, 55)	40 (23, 55)	38 (21, 51)
	GCU	37 (20, 47)	45 (20, 49)	38 (21, 50)	38 (20, 49)	38 (21, 51)	38 (21, 51)
Tyr	UAU	84 (32, 77)	88 (39, 95)	85 (29, 70)	86 (30, 73)	85 (28, 68)	83 (27, 64)
	UAC	16 (6, 15)	12 (5, 13)	15 (5, 12)	14 (5, 12)	15 (5, 12)	17 (5, 13)
His	CAC	13 (4, 9)	9 (2, 6)	15 (4, 9)	17 (5, 11)	17 (5, 11)	16 (5, 11)
	CAU	87 (25, 59)	91 (25, 60)	85 (21, 52)	83 (22, 53)	83 (22, 54)	84 (25, 59)
Gln	CAG	14 (5, 12)	13 (5, 11)	16 (6, 15)	17 (7, 16)	21 (8, 19)	18 (7, 17)
	CAA	86 (32, 76)	87 (31, 75)	84 (32, 78)	83 (32, 78)	79 (30, 73)	82 (31, 75)
Asn	AAC	23 (12, 28)	14 (8, 19)	29 (16, 38)	28 (15, 36)	30 (15, 36)	29 (14, 34)
	AAU	77 (39, 93)	86 (48, 115)	71 (39, 94)	72 (38, 94)	70 (35, 84)	71 (35, 84)
Lys	AAA	83 (88, 211)	86 (106, 257)	81 (72, 176)	81 (72, 176)	80 (67, 162)	83 (59, 142)
	AAG	17 (18, 44)	14 (17, 41)	19 (17, 41)	19 (17, 41)	20 (17, 41)	17 (13, 30)
Asp	GAC	28 (9, 21)	10 (2, 6)	24 (7, 18)	30 (8, 20)	27 (8, 19)	30 (10, 23)
	GAU	72 (22, 53)	90 (24, 57)	76 (23, 56)	70 (19, 47)	73 (22, 52)	70 (22, 53)
Glu	GAA	81 (30, 72)	87 (29, 71)	86 (32, 79)	76 (32, 78)	80 (32, 76)	80 (37, 89)
	GAG	19 (7, 17)	13 (5, 11)	14 (5, 13)	24 (10, 24)	20 (8, 19)	20 (9, 22)
Cys	UGU	81 (12, 30)	81 (11, 26)	97 (14, 33)	86 (12, 30)	91 (13, 31)	94 (13, 30)
	UGC	19 (3, 7)	19 (2, 6)	3 (0, 1)	14 (2, 5)	9 (1, 3)	6 (1, 2)
Trp	UGG	100 (10, 24)	100 (9, 22)	100 (9, 23)	100 (10, 25)	100 (11, 26)	100 (11, 26)
Arg	AGA	29 (28, 68)	40 (31, 74)	36 (38, 93)	34 (36, 89)	31 (34, 82)	33 (34, 82)
	CGG	7 (7, 17)	4 (3, 8)	3 (3, 8)	6 (6, 15)	4 (5, 11)	4 (4, 9)
	CGU	20 (20, 48)	21 (16, 39)	24 (25, 61)	25 (27, 66)	26 (28, 68)	24 (25, 59)
	CGA	26 (25, 60)	18 (14, 33)	27 (29, 70)	25 (27, 66)	27 (29, 70)	26 (27, 64)
	AGG	13 (13, 31)	11 (9, 21)	5 (6, 14)	6 (6, 15)	7 (8, 19)	9 (9, 22)
	CGC	5 (5, 11)	6 (5, 11)	5 (5, 13)	5 (6, 14)	4 (5, 11)	4 (4, 10)
Gly	GGC	17 (11, 26)	7 (4, 9)	8 (5, 13)	8 (5, 12)	9 (6, 15)	8 (6, 14)
	GGU	29 (18, 44)	38 (20, 48)	36 (23, 57)	35 (23, 55)	33 (23, 55)	35 (25, 59)
	GGG	19 (12, 28)	7 (4, 9)	14 (9, 22)	18 (11, 28)	18 (12, 30)	17 (12, 29)
	GGA	35 (22, 52)	48 (26, 62)	41 (27, 65)	39 (25, 61)	39 (27, 65)	40 (29, 69)
Total number of codons		2408	2416	2431	2443	2411	2399

Supplementary table 3. Comparison of substitution rates in Ericaceae.	
Species	Distance to the closest common ancestor of the five species (measured as an average number of substitutions per position in shared protein-coding genes)
Monotropa uniflora	0.320
Hypopitys monotropa	0.139
Arbutus unedo	0.024
Vaccinium macrocarpon	0.08
Pyrola rotundifolia	0.057

Supplementary table 4 - divergence of *accD* and clpP in Ericaceae				
Divergence of accD in Ericaceae				
Amino acid sequence similarity				
	Monotropa uniflora	*Hypopitys monotropa*	*Pyrola rotundifolia*	*Arbutus unedo*
Monotropa uniflora		0,228	0,223	0,242
Hypopitys monotropa	0,228		0,296	0,357
Pyrola rotundifolia	0,223	0,296		0,297
Arbutus unedo	0,242	0,357	0,297	
Nucleotide sequence similarity				
	Monotropa uniflora	*Hypopitys monotropa*	*Pyrola rotundifolia*	*Arbutus unedo*
Monotropa uniflora		0,343	0,386	0,415
Hypopitys monotropa	0,343		0,432	0,444
Pyrola rotundifolia	0,386	0,432		0,426
Arbutus unedo	0,415	0,444	0,426	
Divergence of *clpP* in Ericaceae				
Amino acid sequence similarity				
	Monotropa uniflora	*Hypopitys monotropa*	*Pyrola rotundifolia*	*Vaccinium macrocarpon*
Monotropa uniflora		0,282	0,2	0,206
Hypopitys monotropa	0,282		0,193	0,247
Pyrola rotundifolia	0,2	0,193		0,24
Vaccinium macrocarpon	0,206	0,247	0,24	
Nucleotide sequence similarity				
	Monotropa uniflora	*Hypopitys monotropa*	*Pyrola rotundifolia*	*Vaccinium macrocarpon*
Monotropa uniflora		0,431	0,354	0,345
Hypopitys monotropa	0,431		0,312	0,39
Pyrola rotundifolia	0,354	0,312		0,358
Vaccinium macrocarpon	0,345	0,39	0,358	

Supplementary table 5. Comparison of effect of an alignment tool on a selective pressure estimate for *accD* and *clpP*.

	Percent of codons under negative selection	Percent of codons evolving neutrally	Percent of codons under positive selection	P-value for presence of positive selection
**accD**				
PRANK in the codon alignment mode	67%	32%	1%	0.13
PRANK with TranslatorX	56%	41%	3%	0.087
MUSCLE with TranslatorX	42%	47%	11%	$2*10^{-9}$
MAFFT with TranslatorX	55%	42%	3%	$9*10^{-4}$
ClustalW with TranslatorX	38%	30%	32%	less than 10^{-16}
T-coffee with TranslatorX	48%	47%	5%	$2*10^{-4}$
**clpP**				
PRANK in the codon alignment mode	37%	57%	6%	0.011
PRANK with TranslatorX	43%	51%	6%	0.14
MUSCLE with TranslatorX	36%	49%	15%	0.004
MAFFT with TranslatorX	32%	61%	7%	0.03
ClustalW with TranslatorX	32%	45%	23%	$6*10^{-6}$
T-coffee with TranslatorX	26%	50%	24%	$5*10^{-7}$

Supplementary table 6. Characteristics of libraries, sequencing parameters and output.

Species	mean insert length and insert length standard deviation, bp*	read length, bp	number of reads**	mean plastid genome coverage (minimum coverage, maximum coverage)
Hypopitys monotropa	486±35	2*251	5,814,486	720 (219, 1404)
	5158±885 (mate-pairs libraries)	2*251	6,889,666	
	8705±2537 (mate-pairs libraries)	2*151	7,143,258	
Monotropa uniflora	301±62	2*101	111,913,428	2274 (1115, 4149)
	457±40	2*251	1,542,300	
	2205±325 (mate-pairs libraries)	2*101	9,340,990	
Pyrola rotundifolia	459±63	2*300	4,703,724	442 (25, 1408)
	547±109	2*300	2,246,208	
	5630±1026 (mate-pairs libraries)	2*101	4,140,794	
	5630±1026 (the same library)	2*220	16,687,564	
	5630±1026 (the same library)	2*301	3,504,218	

* - Estimated by mapping reads to the plastomes

** - A read pair counts as two reads. For mate-pairs libraries only properly paired reads count (so-called groups A, B, C in NextClip terms).

Supplementary note 1.
Characteristics of the transcripts of *Hypopitys monotropa* with significant similarity to *accD* and *clpP* and sequences of contigs containing *clpP* and *accD* ORF (ORF are marked by asterisks and sequences are italicized).

accD

Contig name	Length	E-value of a BLASTN match between the *Hypopitys'* plastid *accD* and this transcript.	FPKM	Matching region in the plastome	Commentary
c36330_g1_i5	3577	0.0	33.73	BLASTN alignment to the plastome indicates that this transcript matches to a region with coordinates from 27,598 to 31,174. There are no mismatches with the plastome sequence.	Includes *clpP* and a part of *accD* gene
c36330_g1_i7	3594	0.0	0	BLASTN alignment to the plastome indicates that this transcript matches to a region with coordinates from 27,598 to 31,174. The only difference in an insertion of 17 base pairs, that falls into an intergenic region.	Includes *clpP* and a part of *accD* gene. Seems to be a rare form of the main isoform c36330_g1_i5.
c37588_g2_i1	1230	0.0	32.89	BLASTN alignment to the plastome indicates that this transcript matches to a region with coordinates from 31,405 to 32,634. Includes one single-nucleotide differences from the plastid genome within a tRNA gene.	Includes a part of *accD* and three tRNA genes. Due to a similarity of FPKM with the FPKM of c36330_g1_i5, we suppose that this is a part of the same transcript. The inability of Trinity to assemble them as a whole may originate from, for example, an RNA editing.

c82722_g1_i1	269	10^{-26}	3.12	BLASTN alignment to the plastome indicates that this transcript matches to a region with coordinates from 31,061 to 31,188 with a sequence similarity of 80%. This is a region within *accD*.	We suppose, this is a pseudogene within the nuclear or the mitochondrial genomes. The best match of BLASTN alignment against NCBI NT with default parameters is within a plastid genome of *Actinidia chinensis* isolate AD-6
c95803_g1_i1	122	$2*10^{-23}$	0	BLASTN alignment to the plastome indicates that this transcript matches to a region with coordinates from 30,911 to 30,987 with a sequence similarity of 92%. This is a region within *accD*.	We suppose, this is a pseudogene within the nuclear or the mitochondrial genomes. The best match of BLASTN alignment against NCBI NT with default parameters is within a plastid genome of *Monotropa hypopitys* isolate 2KALR
c60384_g1_i1	294	$3*10^{-21}$	1.89	BLASTN alignment to the plastome indicates that this transcript matches to a region with coordinates from 31,819-31,906 with a sequence similarity of 86%. This is a region within *accD*.	We suppose, this is a pseudogene within the nuclear or the mitochondrial genomes. The best match of BLASTN alignment against NCBI NT with default parameters is within a plastid genome of *Monotropa hypopitys* isolate 2KALR
c136046_g1_i1	125	$9*10^{-15}$	0	BLASTN alignment to the plastome indicates that this transcript matches to a region with coordinates from 30541-30601 with a sequence similarity of 90%. This is a region within *accD*.	We suppose, this is a pseudogene within the nuclear or the mitochondrial genomes. The best match of BLASTN alignment against NCBI NT with default parameters is within a plastid genome of *Monotropa hypopitys* isolate 2KALR

c31896_g2_i1	1361	$4*10^{-6}$	88.38	BLASTN alignment to the plastome returns a short match (35 bp) with a region from 31,852 to 31,886 bp. This is a region within *accD*.	Alignment by BLASTN to NCBI NT indicates the closest match to *Vitis vinifera's* clathrin gene with e-value $3*10^{-128}$ and a similarity of 78%. This is, possibly, a transcript of *Hypopitys'* clathrin, with a random coincidence of its short region to a short part of *accD*.
**clpP**					
Contig name	Length	E-value of a BLASTN match between the *Hypopitys'* plastid *clpP* and this transcript.	FPKM	Matching region in the plastome	Commentary
c36330_g1_i5	3577	0.0	33.73	BLASTN alignment to the plastome indicates that this transcript matches to a region with coordinates from 27,598 to 31,174. There are no differences from the plastome sequence.	Includes *clpP* and a part of *accD* gene.
c36330_g1_i7	3594	0.0	0	BLASTN alignment to the plastome indicates that this transcript matches to a region with coordinates from 27,598 to 31,174. The only difference in an insertion of 17 base pairs, that falls into an intergenic region.	Includes *clpP* and a part of *accD* gene

c61870_g1_i1	367	$7*10^{-25}$	0.74	BLASTN alignment to the plastome indicates that this transcript matches to a region with coordinates from 28,765 to 28,654 with a sequence similarity of 85%. This is a region within *clpP*.	We suppose, this is a pseudogene within the nuclear or the mitochondrial genomes. The best match of BLASTN alignment against NCBI NT with default parameters is within a plastid genome of *Monotropa hypopitys* isolate 1VOLR
c37530_g3_i1	233	$3*10^{-10}$	0	BLASTN alignment to the plastome indicates that this transcript matches to a region with coordinates from 28,497 to 28,536 with a sequence similarity of 98%. This is a region within *clpP*.	We suppose, this is a pseudogene within the nuclear or the mitochondrial genomes. Although the similarity to the plastid *clpP* is high, the length of the matching subsequence is very short (40 bp). The best match of BLASTN alignment against NCBI NT with default parameters is within a plastid genome of *Monotropa hypopitys* isolate 1VOLR
c81526_g1_i1	150	10^{-8}	0	BLASTN alignment to the plastome indicates that this transcript matches to a region with coordinates from 28,787 to 28,536 with a sequence similarity of 74%. This is a region within *clpP*.	We suppose, this is a pseudogene within the nuclear or the mitochondrial genomes. The best match of BLASTN alignment against NCBI NT with default parameters is within a plastid genome of *Monotropa hypopitys* isolate 1VOLR
c67804_g1_i1	165	10^{-8}	0	BLASTN alignment to the plastome indicates that this transcript matches to a region with coordinates from 28,653 to 28,719 with a sequence similarity of 81%. This is a region within *clpP*.	We suppose, this is a pseudogene within the nuclear or the mitochondrial genomes. The best match of BLASTN alignment against NCBI NT with default parameters is within a plastid genome of *Monotropa hypopitys* isolate 1VOLR

c54708_g1_i1	238	$5*10^{-8}$	0	BLASTN alignment to the plastome indicates that this transcript matches to a region with coordinates from 27,982 to 28,030 with a sequence similarity of 88%. This is a region within *clpP*.	We suppose, this is a pseudogene within the nuclear or the mitochondrial genomes. The best match of BLASTN alignment against NCBI NT with default parameters is within a plastid genome of *Monotropa hypopitys* isolate 1VOLR

>c36330_g1_i5 - includes clpP and a part of accD
GAGGCTCTTGCTCAGTCTTATCTTTTGCTTCTGCAGCAAGATGCTCTTGCTCAGCCTTCTC
CCTTTCTTTTATAAGTTCTTTTAAGTTCTCGTGTATCTCCTCAATTATTTCGTCTACCCCAGA
CTCCCATTTAACAAAATCAAAAGGCTTCACTTCTTTTTCATAATCAAATTCAATGGGATCCAG
AGAGAACATGTCCTCATCCATAGGAGACCAAGTGTCTGAATCAACCAAAAAGCCTATTCTA
TCTGAACTATTCATTTGCACATGTGATTGGCACCATTCACAAATATACAGTTTTGAATTAAAA
AGCGATTTATAATTTATTCCATAACAACTTTTGCATTGAACCCACAAATGGCTATAATCGGG
ATGACTCTTATTATCACTGCCCTCTTCATTGCTTTTTTTATCTGGATCTATTTCGTTCTCCTG
ATCTATTTTTTTCTGTTGAAAAGGACCATTATCAGGACCATTATCAGGGTATATTATTTCTCT
TTTATAATATTGCAAGCCGTCCTCTATTTTATTGAGCTTTTTATCTATCTCCTCGTCTTTACTT
TGATCTAATTCGTACTGTAGCTCACTTAATTTAATTTTATTCCTTAATCTCTCTATATCCAGG
GTGTATTGGTCAAAATCCCCCATCGATATCTCCCTGGTTCTCCTTTGATCTAATGCACGCTG
TTGCTTTCTTTCTTTCAGTTGATTCCTTGCACCCTCTAGATCTAGGATGCGTTGTTCTAAATC
TTCTTTAAATCGATCGGTGTCTCTAAAGTAATTGATAACAACACTTAGATGTTCGGATGCCA
GTCCACGGGTTTTATTCTCTAGATCTACTATATCAAAATTTTTATCAAATTTTTTATGTTTCCT
TCTATGATTATCCGACACAGACTCATCCCGCCTCTTGGCATTACCTAAATTTTTAAAATTAG
AGGATATTTGTGTCTGCTTCAATTTACTAAGTTTGTAGCTCCAAATAGCAAAAATACTGAAA
_GGAACTACCCCTATATTGCCGATTAATTTTTTTATTTGCAT_*ACTATATACCTCAGGTGTA
AAATATAATTTTTAATTAAAAATAAAAAGTATGGTAGATTCAAACGAGCGTTCTATC
TAATCGTATGGTTAAGATTTAGTTTTCCCATACAATTAGTTTTCCCATACAATTAGTT
TTCCCATACAATTAATTTAATAATTCGTCCGCATAAACTAAAACTATTAAAAATTTTT
AAGAACCCTTTAAGAATGCGTCCACAGATAACCAAAACCAGACAAGTCCGATATAA
AATAAAACTATTAAAAAAAATTTTACCAACCCTTTTAAAAACCAGATTACTAAAGTC
CAAAAAGATTTATCCATATAGGCTCTCCTAATTTTAATTTTTAATATTTAATAGTAGA
AATAATAGTCTACTTCTACGTTTGACAACAAACATGCCCAGAGAAGATTGAATAAA
GAAAGAATTAAAATTAATATTGGAAATTTTTTTGTCAAATAGGCAAAATAGAATGG
ATCTACTTTATCTATTTTGCAAGCGTGTCCATGGTCGAGAATTACCCAAACTGCTATG
TAATCTTTTACATTACACAGGACAAAGATAATAAGAATTCGCATTATTATGTCAACT
TTCTTGCTCAACAGTTTGAATACTATAAACAGTGGAATATTAAGGAAAAAAGTAATC
ATATAGATTCTATAGAAATATCCTACTTGATCCGTGTTGATAGGGTTAACTTTTTTAA
GATATAAAACTAAAACAATAATAAGAGACTTGAACAAGTCCTTAATATTCCAGTGG
TTAAAGGTAAAACTATTAACGAACTCTCTCTTATAAAATCTTTGAGTTTTATAGTCA
AATTTATGAGTATAGAAGTCGTCTGGTTCTGTGTCCACAAACAACCAAGCCCATACA
AAATTGACTAAACAAGTAATCAAAAAGATTACAGGTAGATTTTTAATTATATAGTTA
GGATCGGCGACTTGGAACAGTTGAAACGAAGTATACTTTTTCAGACCATGGATACAT
ATCCAACCTGCCAGCCAATCTTTGCTAGTCCAAATTGCACAAATAATAGCAGCTCGT
GTTACACTGTTGTTCAGCGGGTTCACGATAAATACCGCTATAACAGTGCAAAAAGTC
ATAACTTTGAGAAGATTCAAGTAATAATAGCTGAGGATATCTACATATAAATAGGA

GGATTTCATATTATGAAACTTTCGCAAATAATAGAATACCGGCCAGATCTTTACCGC
ATCTTTTAGAATACAAAGTAATACTATATCTAATTTAAGCATTTT*_TTATTCCCCATTTT_
TGTCTGATTTTTCCTGGGTTTCATCCTCAGGTTTTTAAAATTAAAACCACGTATGATCTCGT
CGATAATTCCGTACTCTCGGGCTTCTGCCGCGGACATATAACGCACTCTTCTTCCTATATC
TCTTATAAATTCAGGAGGTTGCCCCGTTTTTCCGTAAAAATATCTTGTAGAGATCCACATA
TCTGCAATTTTATGTTTGCATCAACTTCATATTCTGAGGGTGGAAGACGCGGATCCAATTTA
ATGTTCGGTCGAGAAAGCAGGAACGAACAGTTAGGGAATGCTAAACGTGTCCTTCCCCCA
AGCAAGGCATAGATTCCCGGTCCTCCAACCTGCCCGCAGCCTATTGTCTCTATGTTTATGT
CTGACCCCTCATTCATGAAATTACAAAGAGTCCTTGAAGCAAGTAGGCTTCCTTTAGAGCA
GTTTATAAGAACGCTTGCCGTTTCCGTCGCATCACCATAAATATCAAGGCATATTATCAATT
TTATAATTTTATTAGCGACCTCCATAGTGAGATTTTCCCATAGAAATATAATACCGTTCTTAT
AAAGAAAAGAATATAAATCAATAAAATTTACTACTTCTTCATAATATATTTTTTTTTCATAAATT
ATTTCTGCCTCATCCTCATCCTCATCCCCATTTTCACTGTCATTAGGTTCATTTTCAATTCTT
TCTAACTCTTCAACTTCTTCTATTTCTATATAAGGAACTTTAGGGATAATTATTTAGGCTCTT
TAGGCTCTTTTTTCTCCTTCTTCTTCCTTTACCCTTTTTATCCTTTTCATCCCCTTCATCGTC
_AAAAGGATCGTCAAAAGGATCTGCGTTTATGTAATTTCGATAAAAAATCAT_*TTCCTTTTGG
TGTTGTAGTTTGTTGTTTTATTTAGGTGTTTTACCTTGGCAAGTCCCCCTTTTTACCTT
GGTAAGTTCCCCTTTAAGTCTTCTATTAAAAAACTTATATTTTCAATTAAAGGACATC
CAATATTTCAATATACCATATTTTATTTTTATACTTTGTCAAAAGAAGCCCGTCCTCA
CCCGGGCTAGACGCGTACTTATATGGTATGATTGAATTCTAATGATAATCTGGTTAC
GGAGCGATTACTTTATTTTAATCTTTTTATTTGAATATTTTGAATAGTAAAGTTTTCC
AAAAATGGAAAACGAGCAAGCTTGTAAGGTAGATGACTATTGGAGACATTCCTTTT
ATTCCTTTATTTATTAAAATATATGGATATATCCATTTTATA

>c37588_g2_i1 - includes the other part of accD, plus three tRNA genes
CCCGCAGCTTCCGCCTTGACAGGGAGGTGCTCTGACCAATTGAACTCCAATCCCAGG
GAGGCATATATTATGTTTGTCTCAGTTTATAATCTTATGGTATATGGTATATCGGTGA
ATTATTGAGCCGAGCTGGATTTGAACCAGCGTAGACATATTGCCAACGAATTTACAG
TCCGTCCCCATTAACCACTCGGGCATCGACCCGGTAAGAATAATTTTAATTTTTAGA
TTTATTGGTAATCACTGATAAAACTCCTTTATTAGTACTCTACCCCCAGGGGAAATT
GAATCCCCGCTGCCTCCTTGAAAGAGAGATGTCCTGAACCACTAGACGATAGGGGC
ACACACTTGCCCGGCTGCTACTTACATCATACTAAGATCATAGTATAAACTTCTTTTT
GTAACACAAAATATAAAGATATAAAAAAGGGTAATGATAATAAAGTTAAAGGAAC
AATAACCTCTTATCAATTCTATATCTATAAGTATAAGAACAAGAGGTTATATAAATA
TTATATAAATATATAAATTAATTTAACAAAACAGTCTATTTCATTTTCATTGACTTTA
TTTAATAATTGAATACAATTTATCAA*_TTAAGCAAAAGCAAGCCGGTAATTGTGAAAGA_
GATATAGCAAAGACAGAATTCTTTTAAAATACTTACGTGAAAGGATTAGATCGAAGGCGCC
CTTTTGGAATAAATATTCGGTCTCTTGGGCACCCTCAGGGACTTCTATATGCAATGTTTCTT
CAATTACTCTTCTGCCCGCAAATGCAATGACGGCCCCGGGTTCGGAAATAATTACATTCCC
CAACATACCAAAACTAGCTGTCACCCCACCAGTCGTAGGAGATGTAAGGATTGATACATAA
AATAGTTTTTTATCTAATTTATAAGTATATAATGCACAAGCTATTTTACTCATTTGCATCAAGC
TCAAACTCCCTTCTTGCATACGCGCACCCCCAGAAGCACATATTATAACAAGGGGTAGAAA
TTCTTTGATAGCATACTCGATCAAACGGGTAATTTTTTCTCCAACTACGGACCCCATAGTAC
CCGCTATAAACTTAAAATCCATAACCCCAAGTGCTACCGGAATACCCTTTATTTCAGCTATG
CCTGTTTGAATAGCATCATTTAAGCCTGTCTCGCTTTGATAATAATTGAGGCGCTCTTCATA
GGTCTTATCCTTTTCTTCTTCTTCTTCTGCATCAAGATGCTCTTGT
